# A conserved sequence in the small intracellular loop of tetraspanins forms an M-shaped inter-helix turn

**DOI:** 10.1038/s41598-022-07243-y

**Published:** 2022-03-16

**Authors:** Nikolas Reppert, Thorsten Lang

**Affiliations:** Department of Membrane Biochemistry, Life and Medical Sciences (LIMES) Institute, University of Bonn, Carl-Troll-Straße 31, 53115 Bonn, Germany

**Keywords:** Biochemistry, Structural biology

## Abstract

Tetraspanins are a family of small proteins with four transmembrane segments (TMSs) playing multiple roles in human physiology. Nevertheless, we know little about the factors determining their structure. In the study at hand, we focus on the small intracellular loop (SIL) between TMS2 and TMS3. There we have identified a conserved five amino acid core region with three charged residues forming an M-shaped backbone, which we call M-motif. The M´s plane runs parallel to the membrane surface and the central amino acid constitutes the inter-helix turning point. At the second position of the M-motif, in tetraspanin crystal structures we identified a glutamate oriented towards a lysine in the juxtamembrane region of TMS1. Using Tspan17 as example, we find that by mutating either the glutamate or juxtamembrane-lysine, but not upon glutamate/lysine swapping, expression level, maturation and ER-exit are reduced. We conclude that the SIL is more than a short linking segment but propose it is involved in shaping the tertiary structure of tetraspanins.

## Introduction

Tetraspanins comprise a family of small membrane proteins expressed in all multicellular organisms. The human genome encodes 33 family members^1^. They are involved in many cellular processes of either physiological or pathological nature, including adhesion, cell–cell fusion, endocytosis, exosome formation, immune response, migration, neurite navigation, pericellular proteolysis, proliferation, signalling, spreading, trafficking, vascular morphogenesis and remodelling, thrombosis, tumor progression and metastasis, viral and other pathogen entry, and viral release^1^. The basis for this broad range of functions is their capability to form so-called tetraspanin-enriched microdomains (TEMs)^2^, sometimes also referred to as tetraspanin web^3^. The underlying mechanisms include weaker secondary interactions among themselves and stronger primary interactions^4^ with a variety of non-tetraspanins, for instance integrins, members of the immunoglobulin superfamily, and signalling receptors^3,5,6^.

Until recently, studies have concentrated on members that locate to the plasma membrane, a characteristic that has led to their nick name ‘master organizers of the plasma membrane’^7^. However, lately, their intracellular roles have become more and more obvious. Especially their function in extracellular vesicle formation and targeting^8^ is shifting into focus.

**Figure 1:**
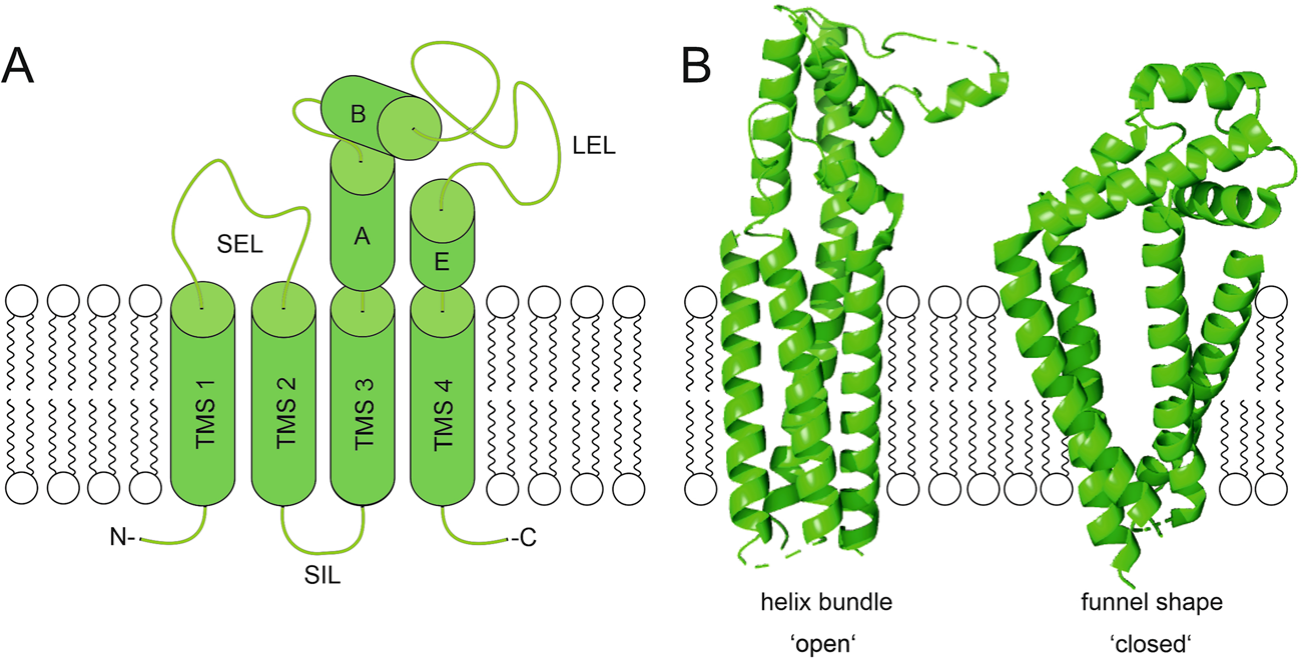
Tetraspanin topology and open-/closed-conformation. Tetraspanin embedded into a membrane bilayer; top and bottom correspond to the extracellular and intracellular side, respectively. (**A**) Schematic depiction of the tetraspanin’s topology including four transmembrane segments (TMS1–4), a small extracellular loop (SEL) and a large extracellular loop (LEL, shown with the conserved helical domains A,B,E), a small intracellular loop (SIL) and a N and C terminus. This illustration was modified from Hochheimer et al.^11^. (**B**) Different conformations proposed for CD81 referred to as helix bundle/’open’ (left, based on PDB: 7JIC) and funnel shape/’closed’ (right, basd on PDB: 5TCX) that differ in the arrangement of the TMSs and the orientation of the LEL; the right structure represents the closed conformation as defined by Zimmerman et al.^12^. The scheme was created using CorelDRAW 2019 (www.corel.com) and the cryo-EM and crystal structure images were created using PyMOL 2.5 (https://pymol.org/2/).

Tetraspanins are membrane anchored via a bundle of four transmembrane segments (TMSs). They share a conserved topology (Fig. 1A) comprised of a small and a large extracellular loop (LEL) connecting extracellularly the first and last TMS pairs, respectively. The LEL, which is often glycosylated, contains up to five helical segments (A–E) and up to four disulphide bridges^9^. With few exceptions, the intracellular N- and C-termini, as well as the small intracellular loop (SIL) connecting TMS2 and TMS3, are short segments^1,10^.

For their tertiary structure, two different models are envisioned. On the one hand, using the crystal structure of the LEL of CD81 as starting point, an early model predicts a tight four transmembrane helix bundle (similar to Fig. 1B, left). The alpha helical structure of TMS3 and TMS4 protrudes and merges with the alpha-helical segments A and E of the LEL, respectively. As a result, the more bulky extracellular domain sits enthroned on top of the bundle^13^; the entire structure resembles a mushroom. In cryo-EM, a closely related open conformation is found for CD81 (Fig. 1B, left) and CD9 in complex with their primary binding partner CD19^14^ and EWI-F^15^, respectively. On the other hand, all known crystal structures of complete tetraspanins (CD81, CD9 and CD53)^12,16,17^ reveal a funnel shaped arrangement of the TMSs opening towards the extracellular site (Fig. 1B, right), with a cholesterol bound inside the cavity of CD81^12^. It is important to note that here the alpha-helical connections between TMS3/4 and the LEL helices are disrupted by a kink, causing the LEL to fold-back onto the membrane, thereby closing the cholesterol cavity. Removal of the cavity-bound cholesterol in molecular dynamics simulation, the connecting segments become more alpha-helical and the LEL unbends, similar to the early model, but the funnel-shaped TMSs arrangement persists^12^. Altogether, the combined structural data result in a model of a conformational switch between an open- and closed-state (Fig. 1B), which modulates the potential interactions and functions of tetraspanins^12,18^. The impact of the switch lies in the reorientation of the LEL that plays a pivotal role in primary interactions^14,19–21^, but the TMSs and intracellular parts are also crucial^16,22–25^. Additionally, a hypothesis claims that the funnel shaped TMS bundle influences membrane curvature generation and/or could locate the protein towards high curvature membrane regions^16^.

The SIL is the smallest segment in human tetraspanins^11^ and may only be a linking stretch between TMS2 and TMS3, with any arbitrary sequence. However, a study found a conservation of 33% between four analysed human tetraspanins and a high conservation of 78% between these human tetraspanins with their homologs in zebrafish^26^. Moreover, Mazurov et al. showed the SIL to be decisive for the interaction of human T cell lymphotrophic virus type 1 gag protein with CD81 and CD82^27^. Apart from that little is known, which makes the SIL a poorly understood tetraspanin section. In this study, we examine whether the SIL is more than just a connecting segment, and identify a conserved SIL core sequence between TMS2 and TMS3. The sequence adopts an M-shape, harbouring at its second position a glutamate. In Tspan17, the glutamate seems to interact electrostatically with a lysine in the juxtamembrane region of TMS1, and by this regulates Tspan17 glycosylation and ER-exit.

## Material and methods

### Cloning of constructs

The tetraspanin sequences of Tspan15 (NM_012339.5), Tspan17 (NM_130465.5), CD53 (NM_001040033.1), CD37 (NM_001774.3), CD82 (NM_002231.4), CD81 (NM_004356.3), CD9 (NM_001769.4) and CD63 (NM_001780.5) were GFP-tagged by cloning the sequences into the pEGFP-C1 vector (Clontech, #6084-1) as described before^11^. EWI-2 (NM_001206665.2) was cloned using the pEGFP-C1 vector without the GFP sequence employing the NEbuilder HiFi DNA Assembly Cloning Kit (NEB, #E5520S). The Myc-tag was fused to EWI-2 at the C-terminus without a linker region. The tag was attached employing two forward primers harbouring the myc tag and a reverse primer complementary to the EWI-2 C-terminus. The used primers are listed below. Tetraspanins were C-terminally fused to monomeric enhanced GFP (mEGFP^11^) with the exception of Tspan17 that is better expressed when N-terminally fused to GFP. For the generation of mutants, we employed Q5 High-Fidelity DNA Polymerase (NEB, # M0491S) and back-to-back primers carrying the desired mutations. The following primers were used (the mutated nucleotides are lowercase): Tspan17-K18A: 5′-gcATACTTCCTGTTTGGCTTCAAC-3′ (fwd) and 5′-CCCGCAGCAGCCGACCTCAG-3′ (rev); Tspan17-K18E, E88K: 5′-gAATACTTCCTGTTTGGCTTCAAC-3′ (fwd) and 5′-CCCGCAGCAGCCGACCTCAG-3′ (rev) then 5′-aaGAACACCTTCCTGCTCAAG-3′ (fwd) and 5′-CCGGAGGGCCCCAATGCAGC-3′ (rev); Tspan17-R87A: 5′-gcGGAGAACACCTTCCTGCTCAAGTTTTTCTCCG-3′ (fwd) and 5′-GAGGGCCCCAATGCAGCCAGC-3′ (rev); Tspan17-E88A: 5′-GcGAACACCTTCCTGCTCAAG-3′ (fwd) and 5′-CCGGAGGGCCCCAATGCAGC-3′ (rev) Tspan17-N89A: 5′-CGGGAGgcCACCTTCCTGCTCAAGTTTTTCTCCG-3′ (fwd) and 5′-GAGGGCCCCAATGCAGCCAGC-3′ (rev); Tspan17-T90A: 5′-CGGGAGAACgcCTTCCTGCTCAAGTTTTTCTCCG-3′ (fwd) and 5′-GAGGGCCCCAATGCAGCCAGC-3′ (rev); Tspan17-F91A: 5′-CGGGAGAACACCgcCCTGCTCAAGTTTTTCTCCG-3′ (fwd) and 5′-GAGGGCCCCAATGCAGCCAGC-3′ (rev); CD9-K11A: 5′-gcATACCTGCTGTTCGGATTTAAC-3′ (fwd) and 5′-GATGCACTTGGTGCCTCCTTTG-3′ (rev); CD9-K11E, E84K: 5′-gAgTACCTGCTGTTCGGATTTAAC-3′ (fwd) and 5′-GATGCACTTGGTGCCTCCTTTG-3′ (rev) then 5′-aAGTCCCAGTGCATGCTGGGAC-3′ (fwd) and 5′-CTGCACAGCCCCGCAGCAGC-3′ (rev); CD9-Q83A: 5′-GGGGCTGTGgcGGAGTCCCAGTGCATGC-3′ (fwd) and 5′-GCAGCAGCCCAGGAAGCCCACCAGCATC-3′ (rev); CD9-E84A: 5′-GcGTCCCAGTGCATGCTGGGAC-3′ (fwd) and 5′-CTGCACAGCCCCGCAGCAGC-3′ (rev); CD9-S85A: 5′-ATGCTGGGACTGTTCTTCGGCTTCCTCTTGG-3′ (fwd) and 5′-GCACTGGgcCTCCTGCACAGCCCCGCAGC-3′ (rev); CD9-Q86A: 5′-GAGTCCgcGTGCATGCTGGGACTGTTCTTCGGC-3′ (fwd) and 5′-CTGCACAGCCCCGCAGCAGC-3′ (rev); CD9-C87A: 5′-GAGTCCCAGgcCATGCTGGGACTGTTCTTCGGC-3′ (fwd) and 5′-CTGCACAGCCCCGCAGCAGC-3′ (rev); CD53-K7A: 5′-gcACTGCTGAAGTATGTCCTG-3′ (fwd) and 5′-CAAGCTACTCATGCCCATGC-3′ (rev); CD53-K7E, E77K: 5′-gAACTGCTGAAGTATGTCCTG-3′ (fwd) and 5′-CAAGCTACTCATGCCCATGC-3′ (rev) than 5′-AAGaAAACAAGTGTCTGCTTATGTCGTTCTTC-3′ (fwd) and 5′-GATAGAGCCCATGCAGCCCAGGAAGGC-3′ (rev); CD53-K10A: 5′-gcGTATGTCCTGTTTTTCTTCAACTTGC-3′ (fwd) and 5′-CAGCAGTTTCAAGCTACTCATGCC-3′ (rev); CD53-K10E, E77K: 5′-gAGTATGTCCTGTTTTTCTTCAACTTGC-3′ (fwd) and 5′-CAGCAGTTTCAAGCTACTCATGCC-3′ (rev) then 5′-AAGaAAACAAGTGTCTGCTTATGTCGTTCTTC-3′ (fwd) and 5′-GATAGAGCCCATGCAGCCCAGGAAGGC-3′ (rev); CD53-K76A: 5′-gcGGAAAACAAGTGTCTGCTTATGTCGTTCTTC-3′ (fwd) and 5′-GATAGAGCCCATGCAGCCCAGGAAGGC-3′ (rev); CD53-E77A: 5′-AAGGcAAACAAGTGTCTGCTTATGTCGTTCTTC-3′ (fwd) and 5′-GATAGAGCCCATGCAGCCCAGGAAGGC-3′ (rev); CD53-N78A: 5′-gcCAAGTGTCTGCTTATGTCG-3′ (fwd) and 5′-TTCCTTGATAGAGCCCATGC-3′ (rev); CD53-K79A: 5′-AAGGAAAACgcGTGTCTGCTTATGTCGTTCTTC-3′ (fwd) and 5′-GATAGAGCCCATGCAGCCCAGGAAGGC-3′ (rev); CD53-C80A: 5′-AAGGAAAACAAGgcTCTGCTTATGTCGTTCTTC-3′ (fwd) and 5′-GATAGAGCCCATGCAGCCCAGGAAGGC-3′ (rev); Tspan15-D87A: 5′-GcCAACCTGTACCTTCTCCAAGC-3′ (fwd) and 5′-ACGGAGGGACGCCAGCACACCAATG-3′ (rev); CD37-E82A: 5′-GcGCTCCGCTGCCTCCTGGGCC-3′ (fwd) and 5′-CTTGAGGGCCCCCACACAACCC-3′ (rev); CD82-E80A: 5′-GcGGTCCGCTGCCTGCTGGGGC-3′ (fwd) and 5′-GTTGACGGCGCCGATGCAGCCC-3′ (rev); CD81-E86A: 5′-cATCCCAGTGCCTGCTGGGGACGTTC-3′ (fwd) and 5′-CCTGGATGGCCCCGTAGCAGCCCAGG-3′ (rev); CD63-E78A: 5′-GcGAACTATTGTCTTATGATCAC-3′ (fwd) and 5′-CTTGCAGGCCCCGCAGCAGCC-3′ (rev); EWI-2 C-terminal Myc-tag: 5′-TCTGAAGAAGATCTGtaaagcggccgcgactctag-3′ (fwd), 5′-GAACAAAAACTTATTTCTGAAGAAGATCTGtaaag-3′ (fwd) and 5′-CCGTTTTCGAAGCCTCTTCATGAAGCAGCAAGTG-3′ (rev). The PCR products were run on a 1% agarose (Carl Roth, #2267.4) gel in TAE (40 mM Tris, 1 mM Na_2_EDTA, 0.1% acetic acid, pH 8.3). The wanted bands were cut out and cleaned up using the Monarch DNA Gel Extraction Kit (NEB, #T1020L). The linear plasmid was phosphorylated using T4 Polynucleotide kinase (NEB, #M0201S) and ligated by T4 DNA Ligase (NEB, #M0202S). The protein coding sequence was verified by sequencing (Eurofins GATC Biotech GmbH).

### Alignment of amino acid sequences and obtaining the consensus sequences

All alignments were done with BioEdit^28^ v7.0.5 (http://www.mbio.ncsu.edu/BioEdit/bioedit.html). The tetraspanins' SIL sequences were aligned with the most N-terminally glutamate or aspartate and if there was none the sequences were aligned at the most N-terminally lysine or arginine. The sequences without a glutamate, aspartate or lysine and arginine were aligned due to overall similarity to the other sequences. The tetraspanins' N-terminal sequences with the first five amino acids of the TMS1 were aligned at their most C-terminal lysine or arginine.

The consensus sequences of SIL and N-terminus were obtained by counting the frequency of each amino acid at the given position. An amino acid was counted as consensus, if its frequency (f) was equal to or higher than the mean frequency (f_mean_) plus its standard deviation (f ≥ f_mean_ + SD).

For the Claudin SIL sequence alignment we used the ClustelW multi alignment tool of BioEdit.

### Structural predictions and depiction

The TMSs of each tetraspanin^11^ were defined using TMHMM Server v. 2.0^29,30^ (http://www.cbs.dtu.dk/services/TMHMM/) and their domains such as N-terminus and SIL were defined as N-terminally of TMS1 and the linker between TMS2 and TMS3, respectively. The secondary structures were analysed by Jpred4^31^ (http://www.compbio.dundee.ac.uk/jpred/). The helices containing TMS1 of each human Tspan were analysed by HeliQuest^32^ (https://heliquest.ipmc.cnrs.fr/cgi-bin/ComputParams.py). The images of crystal and cryo-EM structures as well as the dihedral angles were obtained using PyMOL 2.5 (https://pymol.org/2/). The orientation of the SIL towards the membrane was adopted from the Orientations of Proteins in Membranes (OPM) database (https://opm.phar.umich.edu/). The illustration of amino acid frequency was done via Weblogo^33^ 3.7.4 (https://weblogo.berkeley.edu/logo.cgi). The data composition for the figures was done employing CorelDRAW 2019 (www.corel.com).

### Confocal microscopy

HepG2 cells (ATCC, #HB-8065) were grown in MEM Eagle (Pan Biotech, # P04-08509) with 10% FBS (Pan Biotech, # P303031), 1% penicillin–streptomycin (Pan Biotech, #P06-07050) and 1% stabile glutamine (Pan Biotech, # P04-82100) up to 80% confluence. The cells were detached using trypsin (Pan Biotech, #P10-0231SP) and diluted in DPBS (Gibco, #14190-094) to 14.4 million cells per ml. The cells were transiently transfected with the above described vectors using the Neon transfection system (Thermo Fisher Scientific, #MPK10096) with settings 1200 V, 50 ms, 1 puls. The cells were stained, imaged and analysed essentially as described before^11^. Briefly, the cells ER was visualized by a KDEL-RFP fusion construct (BacMam 2.0, Life Technologies, #C10591) and the signal was enhanced using a RFP-Booster Atto594 (Chromotek, #rba594). Cells were imaged in the confocal mode using a 4-channel easy3D superresolution STED optics module (Abberior Instruments) coupled to an Olympus IX83 confocal microscope (Olympus, Tokyo, Japan), equipped with an UPlanSApo 100x (1.4 NA) objective (Olympus, Tokyo, Japan). GFP was excited with a 485 nm laser and recorded with a 500–520 nm filter. Atto594 was excited with a 561 nm laser and recorded with a 580–630 nm filter. The pixel size was set to 25 nm. The Pearson correlation coefficient (PCC) between the protein of interest and the ER was calculated with a custom made macro using Fiji-ImageJ^34^ (www. https://imagej.net/).

### Tunicamycin assay

HepG2 cells were treated 4 h after transient transfection with tunicamycin (Sigma-Aldrich, #T7765) up to a final concentration of 5 µg/ml in MEM Eagle with 10% FBS. After 18 h of incubation, cells were scraped off and washed with ice cold DPBS and lysed in 200 µl RIPA buffer (Santa Cruz, #sc-24948). After the cell debris was removed by centrifugation for 20 min at 16,000 g the lysate was mixed with 4 × Lämmli buffer with beta-mercaptoethanol (250 mM Tris HCl, 8% (w/v) SDS, 40% glycerol, 20% beta-mercaptoethanol, bromophenol blue, pH 6.8). The samples were heated for 30 min at 37 °C and subsequently analysed via western blot.

### Co-immunoprecipitation assay

HepG2 cells were transiently transfected with equal amounts of vector DNA (6 µg of each plasmid per transfection) of the CD9-mEGFP variants and the EWI-2-myc construct. For each condition, the cells of two transfections (equals to 3.6 million cells) were pooled and seeded. After 22 h of incubation, cells were scraped off in ice cold DPBS, lysed in lysis buffer (150 mM NaCl, 5 mM MgCl_2_, 25 mM HEPES containing 1% CHAPS (Sigma-Aldrich, #C5070-5G)) and subjected to co-immunoprecipitation with GFP-Trap beads (Chromotek, #gta-20) following the manufacturers protocol (1 h at 4 °C incubation with beads, protein was dissociated from the beads by boiling in 2 × Laemmli + 20% β-mercaptoethanol). The Co-IP samples were analysed by Western blot.

### Western blot

The cells were scraped off in ice cold DPBS and lysed 30 min in 200 µl RIPA buffer (Santa Cruz, #sc-24948). The SIL core and lysine mutations of Tspan17 and CD9 were lysed directly in 250 µl 2.5 × Lämmli buffer (156 mM Tris HCl, 5% (w/v) SDS, 25% glycerol, pH 6.8). After a 20 min spin down of the insoluble cell debris, the lysate was mixed with 4 × Lämmli buffer with or without 20% β-mercaptoethanol and incubated for 30 min at 37 °C (CD53) or 5 min at 95 °C (all others). The proteins were separated by a 10% SDS PAGE and blotted onto a nitrocellulose membrane (BIO-RAD, #1620112) in ice cold towbin buffer (25 mM Tris HCl, 192 mM glycine, 20% methanol, pH 8.3). The membrane was blocked using Intercept blocking buffer (Li-Cor, #210218) and incubated with primary antibodies in blocking buffer with added 0.05% Tween-20 (Carl Roth, # 9127.1) over night at 4 °C. The used primary antibodies were rabbit polyclonal anti-GFP (Thermo Fisher Scientific, #A-11122) diluted 1:2000, mouse monoclonal anti-beta-actin (Cell Signalling, #3700) diluted 1:10,000, goat anti-EWI-2 (R&D systems, #A3117) diluted 1:500 and mouse anti-CD9 (Merck, #CBL162) diluted 1:1000. The secondary antibodies donkey anti-mouse coupled to IRDye 680RD (Li-Cor, #926-68072) and donkey anti-rabbit coupled to IRDye 800CW (Li-Cor, #926-32213) were diluted 1:10,000 in blocking buffer with added 0.05% Tween 20. For the Co-IP the secondary antibodies donkey anti-goat coupled to IRDye 800CW (Li-Cor, #926-32214) and donkey anti-rabbit coupled to IRDye 680RD (Li-Cor, #926-68073) or donkey anti-rabbit coupled to IRDye 800CW (Li-Cor, #926-32213) were used. The blots were imaged using an Odyssey Classic Imaging System (Li-Cor) and the images were analysed using Fiji-ImageJ^34^.

### Statistical analysis

All experiments were performed at least three times independently. Microscopy data were averaged per day. Data were analysed using a two tailed and paired t-test or a repeated measures ANOVA. The analysis and illustration of data was performed using GraphPad Prism version 6.04 for Windows (www.graphpad.com). Results are expressed as mean ± standard deviation. Statistical significance was determined (*P < 0.05, **P < 0.01, ***P < 0.001, ****P < 0.0001).

## Results

### Definition of the SIL core sequence

**Figure 2:**
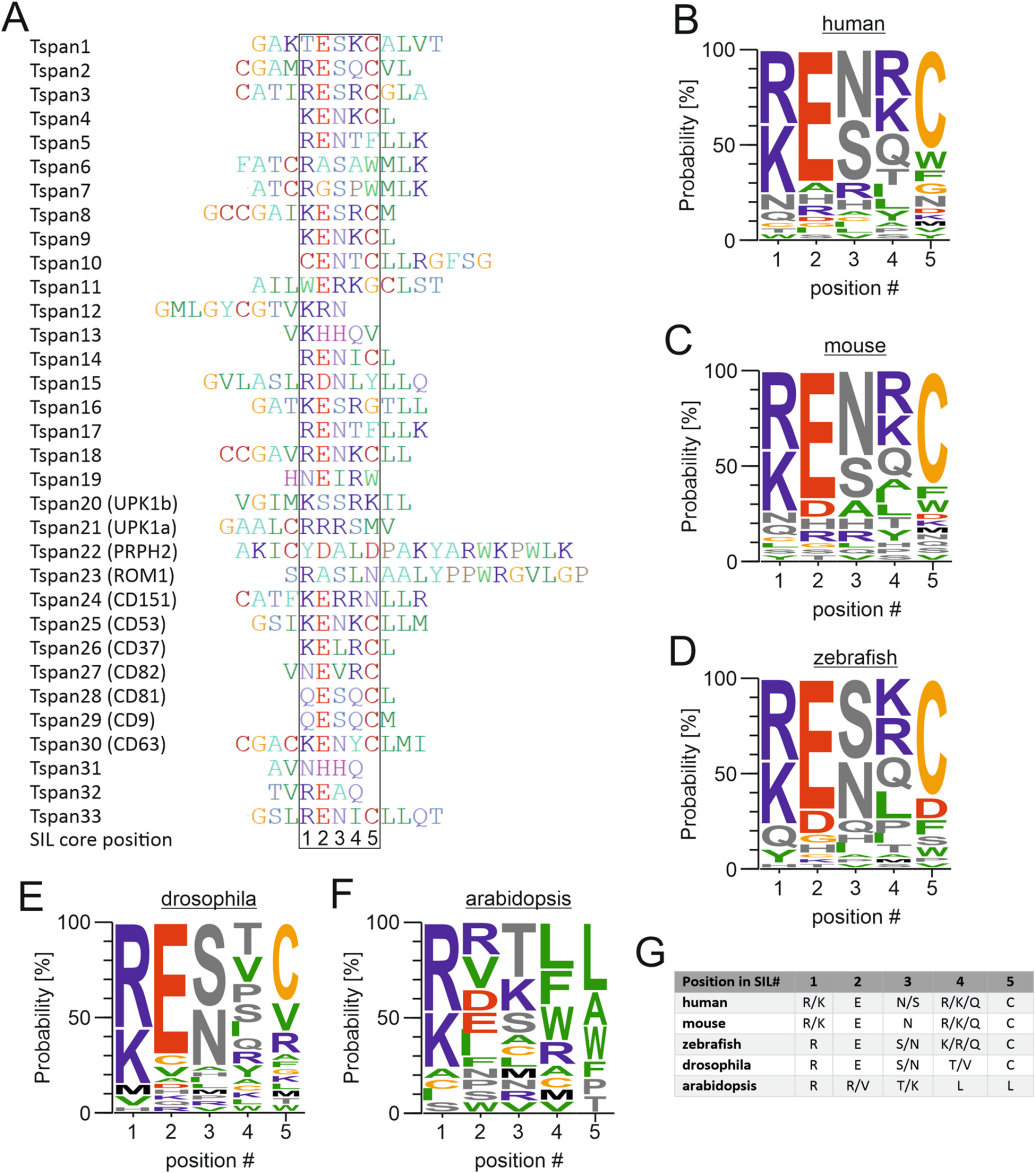
The SIL core sequence. (**A**) Alignment of the SILs from human tetraspanins (SILs segments are defined as in Hochheimer et al.^11^), as described in text, yields an amino acid core sequence with five positions (box). Tspan31 did not feature any of the alignment criteria and was aligned with reference to its similarity to Tspan13. In Tspan12, Tspan31 and Tspan32 some of the positions 4 and 5 are empty, as these amino acids are supposed to be integral part of TMS3. For the SIL alignments of other species see Fig. S1. (**B**–**F**) Residue probability in the SIL core of human (*Homo sapiens*), mouse (*Mus musculus*), zebrafish (*Danio rerio*) drosophila (*Drosophila melanogaster*) and Arabidopsis (*Arabidopsis thaliana*) tetraspanins. (**G**) SIL core consensus sequences. Part of the consensus sequence are amino acids occurring with a frequency f equal or higher than the mean frequency plus one time the frequencies standard deviation (f ≥ f_mean_ + SD). In cases of more than one, they are listed in order of abundancy from high to low. The alignment was created using BioEdit^28^ v7.0.5 (http://www.mbio.ncsu.edu/BioEdit/bioedit.html) and the illustration of residue frequency was done using Weblogo^33^ 3.7.4 (https://weblogo.berkeley.edu/logo.cgi).

In human Tetraspanins, the shortest and longest SIL sequences comprise six and 21 amino acids, respectively^11^. Comparing these sequences, we frequently find a positively charged amino acid directly followed by a negatively charged one, to which we assigned ‘position 1’ and ‘position 2’ of the core sequence (Fig. 2A). We started the alignment of the 33 tetraspanins with position 2, assigning to it the most upstream glutamate (in 23 SILs) or aspartate (2 SILs). In eight SILs there is neither a glutamate nor an aspartate present, which is why we used position 1 for further alignment, finding altogether 24 matches (13 arginines and 11 lysines). Finally, we searched for frequently occurring amino acids in positions 3–5 (Fig. 2A; for details see figure legend). As a result, we identified a [R/K] E [N/S] [R/K/Q] C core sequence (Fig. 2B). With the exception of glutamine at positon 4 that is a polar amino acid, the chemical signature of the sequence is positive charge—negative charge—polar—positive charge—polar.

To compare the human SIL with other species we determined the SIL consensus sequences, as it was done for human, from Uniprot database sequences in mouse (31 family members in *Mus musculus*^11^), zebrafish (50 in *Danio rerio*^35^, but only 39 available in the Uniprot database), fruit fly (34 in *Drosophila melanogaster*^36^) and arabidopsis (17 in *Arabidopsis thaliana*^37^). We obtained the following sequences: [R/K] E N [R/K/Q] C (mouse), R E [S/N] [K/R/Q] C (zebrafish), R E [S/N] [T/V] C (fruit fly) and R [R/V] [T/K] L L (Arabidopsis) (Fig. 2C–F).

Hence, with the exception of plant tetraspanins (Fig. 2F), the degree of conservation between the SILs of all animal species is high (see also Fig. 2G). The question arose whether the same SIL core region is present in similarly structured proteins. Members of the claudin family have four TMSs and two extracellular loops^38^ as well. However, they exhibit a longer and differently structured SIL between TMS2 and TMS3, harbouring a short beta-strand (Fig. S2).

Following, the secondary structure of the SIL core sequences was predicted employing Jpred4. In animal SILs, alpha-helicity gradually decreases from the edges to the central position (Fig. S3). Moreover, it should be noted that the extraordinarily long human SILs in Tspan22 (21 aa) and Tspan23 (19 aa) contain a non-helical stretch with a YXXΦ internalization motif outside the core region, pointing towards possibly additional specialized roles of these two SILs.

**Figure 3:**
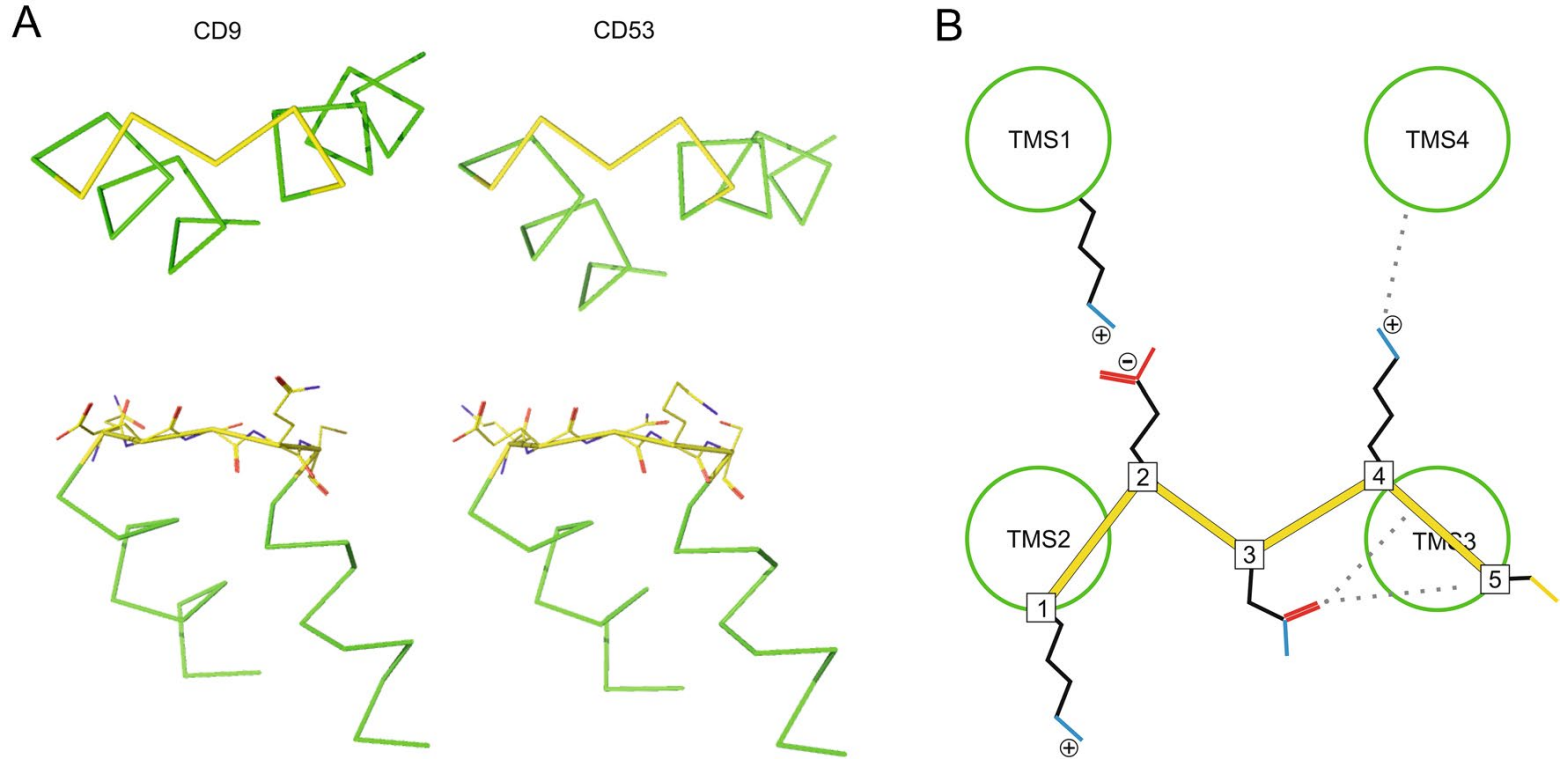
The SIL core forms an M-motif. (**A**) Illustration of the M-motif (yellow) and attached helical structure (green) based on the published crystal structures of CD9 (PDB 6K4J; left) and CD53 (PDB 6WVG; right). Upper panels, top view from the intracellular side. Lower panels, side views. TMS2 (left) and TMS3 (right) are connected to the M-motif and form with the other two TMSs a membrane-embedded funnel (for the complete structures see Fig. S4). The symmetric embedding of the funnel (see OPM database) aligns the M-plane parallel to the membrane. The side view illustrates that the M-motif backbone bonds are co-planar and that, with the exception of position 4, all residues lay roughly in the M-plane. (**B**) Summary of interactions between consensus sequence amino acids that were identified in the crystal structures of CD9 and CD53. Position 2: salt-bridge between glutamate and lysine of the N-terminus in CD9 (for sequence alignment of the N-termini see Fig. S6); in CD53 similar arrangement but distance is too long for a salt-bridge (for details see Fig. S4). Position 3: interactions between the carbonyl-group of an asparagine with the backbone of TMS3 in CD53 (Fig. S4). Position 4: interaction between the lysine side chain and the backbone of TMS4 in CD53 (Fig. S4B). In the stick representation of the side chains, red, blue and yellow indicate oxygen, nitrogen and sulphur, respectively. ⊕/⊖ indicate amino acid residue charges at physiological pH. The scheme was created using CorelDRAW 2019 (www.corel.com) and the crystal structure images were created using PyMOL 2.5 (https://pymol.org/2/).

In the crystal structures of CD9 and CD53, albeit different on the level of amino acids (QESQC versus KENKC; note that for crystallographic reasons in the CD53 structure cysteine is exchanged by a serine), the SIL core structures are essentially identical. The bonds defining the SIL backbone are coplanar, forming an “M”, while the two bottom M-endings mark the transition to the helical structure (Fig. 3A). The non-helical central polar residue (position 3) not only marks the turning point of the protein backbone, but also interacts with the backbone and residues of TMS3 (Fig. 3B). The glutamate (position 2) and glutamine/lysine (position 4) residues are oriented towards TMS1 and TMS4, respectively, whereas the amino acid residues at positions 1 and 5 are not oriented towards any of the TMSs. With the exception of position 4, all residues are roughly lying in the “M” plane (Fig. 3A, bottom) that runs parallel to the membrane surface. In CD9, the glutamate at position 2 forms a salt-bridge with a lysine (K11) of the N-terminus (Fig. S4A), and in CD53, the lysine at position 4 interacts with an asparagine (N207) within the C-terminus (Fig. S4B).

**Figure 4:**
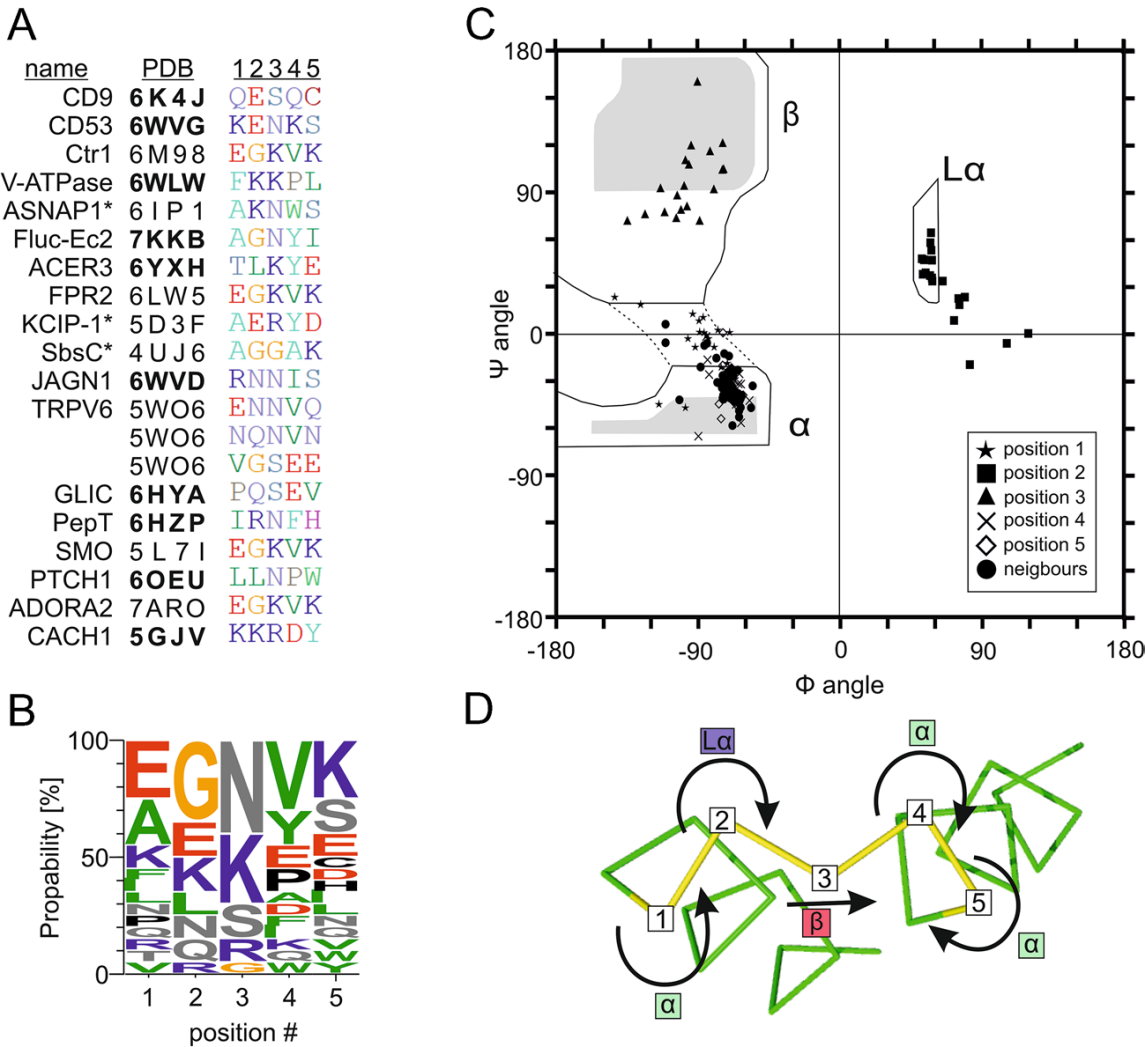
The M-motif is a common structural element formed independent of the SIL consensus sequence. (**A**) The two known tetraspanin structures with resolved M-motif and additional 16 examples of crystal structures containing in total 20 inter-helix turn M-motifs. Proteins are listed using short names, followed by the PDB entry number and the sequence of the M-motif. Mostly, the M-motif was found in membrane proteins but also in three cytosolic ones (marked with an asterisk). Examples in which the motif connects two TMSs were highlighted (PDB number in bold). For structural illustration of the M-motifs in the high-affinity copper transporter Ctrl1, V-ATPase and alpha-soluble NSF attachment protein see Fig. S5. (**B**) Amino acid frequencies in the M-motifs of the proteins listed in A. With the exception of the central asparagine, there is hardly overlap with the animal SIL core consensus sequence. (**C**) Analysis of the dihedral angles employing a Ramachandran plot reveals alpha-helical properties in positions 1, 4 and 5, left-handed helix in position 2, and beta-strand features in position 3. As expected, neighbouring residues are essentially alpha-helical. (**D**) The 180° turn of the direction of the protein backbone involves three structural elements. The turn is initiated by the left-handed character adopted by position 2. Between position 2 and 4 the gap is spanned by the beta-strand character of position 3. Finally, at position 4 already alpha-helicity in the opposite direction is realized. Please note that arrows shown at position 2 and 4 look identical but indicate different angle combinations, which is due to simplification in structure presentation. The alignment was created using BioEdit^28^ v7.0.5 (http://www.mbio.ncsu.edu/BioEdit/bioedit.html) and the illustration of residue frequency was done using Weblogo^33^ 3.7.4 (https://weblogo.berkeley.edu/logo.cgi). The presentation of the dihedral angles was done using GraphPad Prism version 6.04 for Windows (www.graphpad.com) and PyMOL 2.5 (https://pymol.org/2/).

This raised the question if the M-motif is a structural element for which the SIL consensus sequence is a prerequisite. For verification, we screened the PDB database for other α-helix rich proteins exhibiting M-motifs. We readily found more examples (Fig. 4A) from which the detailed structure of a selection is shown in Fig. S5, that however have a sequence very different from the SIL consensus sequence (Fig. 4B). The only overlap is the asparagine at position 3, which is involved in interactions between the side chain carbonyl group and the backbone of the C-terminally attached helix in tetraspanins and non-tetraspanins. On the other hand, when measuring the φ and ψ angles in the M-motif, there was clear position-dependent segregation into φ/ψ angle categories. Amino acids in positions 1, 4 and 5 adopt angles typical for alpha helices, in position 2 we find angles associated with a left-handed helix, and for position 3 the angles are typical of a beta-strand (Fig. 4C)^39^. These specific dihedral angles are the basis of the M-motifs shape (Fig. 4D).

### Crosstalk between the SIL and the N terminus

As outlined above, the SIL could be simply linking TMS2 and TMS3. On the other hand, it might influence the tertiary structure. Of particular significance for stabilizing the protein could be a salt bridge between the SIL glutamate and the N-terminal lysine, such as seen in the CD9 crystal structure (Fig. S4A).

We had some preliminary data pointing towards the functional importance of the SIL in Tspan17. As for Tspan17 no crystal structure is available, we used another type of analysis to predict whether crosstalk between the SIL glutamate and the N-terminal lysine is possible. The N terminus constitutes of 19 amino acids with two lysines at positions 4 and 18. An analysis of the TMS1 N-terminal sequence shows that the positive charge, mostly provided by a lysine, is highly conserved across all animal species (Fig. S6). Based on this conserved position in the alpha helix, relative to a previously described conserved asparagine^12,13^, the lysine of Tspan17 and most other tetraspanins is likely oriented towards the middle of the four helix bundle, and consequently towards the SIL (Fig. S7). This suggest that some crosstalk between the SIL and the N-terminus is possible.

To test experimentally the hypothesis that the SIL interacts with the N-terminus, we analysed the expression levels of Tspan17 after mutating single SIL core amino acids to alanine (Fig. S8), including for comparison CD9 and CD53 as well. With the exception of CD53, we observed strongest diminishment of expression after mutation of the SIL glutamate at position 2.

**Figure 5:**
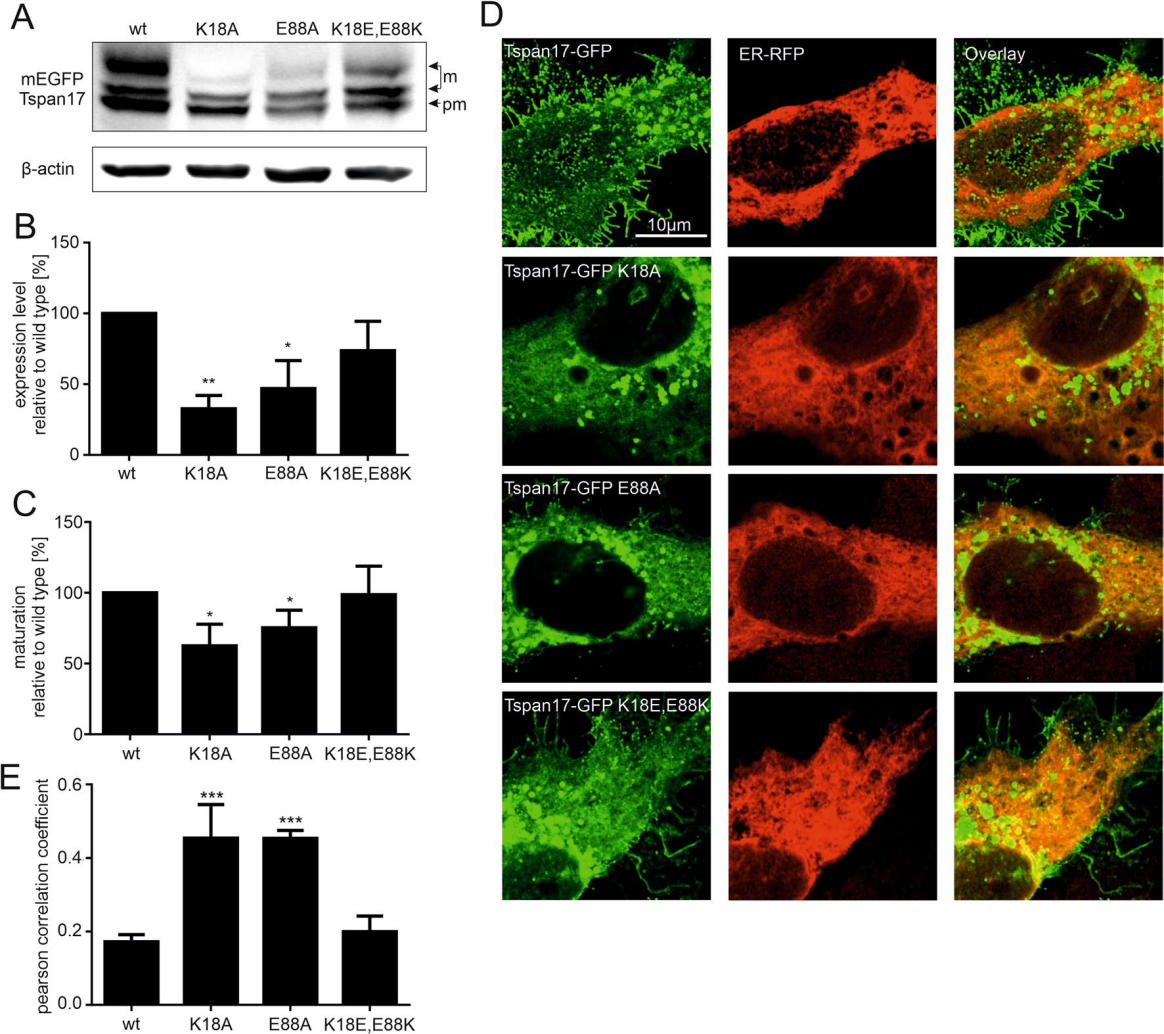
Analysis of the glutamate-lysine interaction*.* (**A**) In mEGFP-Tspan17, glutamate or lysine were exchanged to alanine (mutants K18A and E88A) or interchanged (K18E/E88K). Constructs are expressed in HepG2 cells, and expression levels are analysed by Western Blot analysis. Maturation of Tspan17 involves N-glycosylation and the different bands representing pre-mature (pm) and matured (m) protein are marked with an arrow (for identification of the bands see Fig. S9). (**B**) The actin normalized GFP signal shows a drop in expression level upon glutamate or lysine mutation (wild type values are set to 100%). The mutation with interchanged charged residues restores the expression level. The drop in Tspan17 expression and maturation is detectable shortly after expression starts (Fig. S10). (**C**) The maturation was calculated as ratio between mature to total protein and was compared to the wild type (set to 100%). The glutamate-/lysine-mutation leads to a drop in protein maturation, whereas the double mutation has no effect. (**D**) The co-localization of the mEGFP-Tspan17 constructs with the ER were analyzed by confocal microscopy, comparing the distribution of the GFP signal to an ER marker fused to RFP. For quantification, the Pearson correlation coefficient between the two channels was calculated. Exemplary images of Tspan17 wt and the mutants are shown. (**E**) The Pearson correlation coefficient showed an increase of ER co-localization for the glutamate-/lysine-mutants and no effect of the double mutation. Values are given as means ± SD (n = 4 for Western blot analysis and n = 3 for microscopy; for each biological replicate 10 cells were imaged). The statistical analysis was done employing a repeated measures ANOVA comparing each mutation with the wild type. The full blots are shown in the supplementary data (Fig. S20). The data analysis and illustration was performed using Fiji-ImageJ^34^ (https://imagej.net/) and GraphPad Prism version 6.04 for Windows (www.graphpad.com), respectively.

If the SIL glutamate interacts electrostatically with the N-terminal lysine, exchanging in Tspan17 the lysine to alanine should affect expression just like the glutamate mutation. Because reduced expression levels can have many explanations, in the following we included as well the analysis of glycosylation by western blot and ER-exit by microscopy. In the latter assay, retention in the ER is revealed by an increase in overlap between Tspan17 and an ER-marker. As shown in Fig. 5, mutation of either the SIL glutamate or the N-terminal lysine reduces expression and glycosylation and causes ER retention. Next, we exchanged the positions of glutamate and lysine, which may neutralize the effect of the single mutations as the putative electrostatic interaction may work as well with exchanged positions of the charges. As shown in Fig. 5, all effects are back to normal in the double mutant.

The same mutations in other tetraspanins, e.g. CD9, yield the same expression pattern as for Tspan17 (Fig. S11A) but no effect on ER-exit (Fig. S12). Because in our assay we detect no change in the CD9 band pattern after tunicamycin treatment (Fig. S9), we did not employ maturation analysis of CD9 via probing its glycosylation status. Instead, we examined whether the interaction with the primary binding partner EWI-2 is affected. As shown in Fig. S13, single mutations precipitated more EWI-2 than the wild-type or the double mutant. Finally, in CD53, single mutations had no effect on expression and maturation (Fig. S11B), although the double mutation drastically diminished expression.

Altogether, the data across different tetraspanins is not consistent, in particular not between CD9 and CD53. Trying to understand better the different roles of the glutamates in CD9 and CD53, we took a closer look at the crystal structures. The short N-termini (12 residues) of CD9 and CD53 contain three and two positively charged amino acids, respectively. In CD9, as already mentioned above, the SIL core E84 forms a salt bridge (distance 2.8 Å) with the second last amino acid of the N-terminal peptide (K11) (for illustration see Fig. S4A). In CD53, although the SIL core E77 is oriented towards K7 and K10 of the N terminus, the distances are about one angstrom too long to establish a salt bridge (Fig. S4B; K7-E77, 5.1 Å and K10-E77, 5.2 Å instead of 4 Å required to form a salt bridge^40^). This suggests a weaker or no electrostatic interaction between the CD53 SIL and the N-terminus and could explain the lacking effect in the CD53 mutants.

## Discussion

### The SIL core sequence

The tetraspanin sequence analysis of the SIL between TMS2 and TMS3 reveals a conserved [R/K] E [N/S] [R/K/Q] C core sequence in human, similar to mouse and zebrafish. In fruit fly, the positive charge in position 4 is lacking, and in arabidopsis the sequence is very different. In proteins with the same topology, as in the family of claudins, the SIL is longer (Fig. S2), has a longer unstructured stretch with a predicted beta-strand, is more diverse, and exhibits no similarity to the tetraspanin core sequence. This may point to a specific function of the SIL in mammalian tetraspanins.

It is known that positively charged residues close to the cytosolic site of a TMS are beneficial for its membrane insertion, whereas negatively charged or polar residues decrease the TMS insertion^41^, known as the “positive-inside rule” and the “negative inside depletion/outside enrichment rule”^42^. Therefore, the presence of positively charged amino acids in the SIL is not surprising, as it aids the correct insertion of the nascent protein into the ER membrane^43^. The negatively charged glutamate neutralizes one positive charge. Apparently, this is not relevant for expression, as in CD9 and Tspan17 mutants with swapped glutamate/lysine express equally well as wild-type (Fig. 5, Fig. S11A).

Apart from that, tetraspanins are known to be palmitoylated at several intracellular cysteine-residues, among them cysteines in the SIL of CD9 and CD81^44^, explaining the abundancy of cysteines at the end of the SIL core region (Fig. 2G).

### The SIL forms an M-motif

In animal tetraspanins, the three central residues of the SIL core region are predicted to be less helical, which is consistent with the crystal structures of CD53 and CD9. Please note that a crystal structure of CD81 is also available, but could not be used for detailed SIL analysis as the 2nd and 3rd amino acids of the SIL core are unresolved.

In all animal tetraspanins, the non-helical part of the SIL is on average 2.1 amino acids in length (see also Fig. S3), which is close to the shortest possible linker between two TMSs, that is two amino acids^45^. Roughly speaking, the five core residues form a U-turn with helical arms continued by the TMS helices.

The amino acids at positions 2 and 4 constitute the upper two tips of the M-motif and their residues point towards TMS1 and TMS4, respectively. The position 1, 3, and 5 define the lower three tips of the M-motif, all pointing away from the centre of the TMS-bundle. The M-motif shape is not exclusive to tetraspanins but found in many other soluble and membrane proteins (Fig. 4, Fig. S5), although the amino acid sequence is different from the SIL core sequence, with the exception of the central asparagine.

The left-handed character of position 2 and the beta-strand character of position 3 define the starting point and the bridge of the U-turn (Fig. 4D). Moreover, they are involved in stabilizing interactions as shown by frequent examples for the residue at position 3 that interacts with the backbone of the C-terminal helix (e.g. CD9, PDB: 6K4J and POT family transporter, PDB: 6HZP) or forms a salt-bridge with position 1 (e.g. adenosine A2A receptor, PDB: 7ARO or Smoothened, PDB: 5L7I). Additionally, the residue of position 2 can form salt-bridges with adjacent structures (e.g. CD9, PDB: 6K4J or voltage-gated calcium channel Cav1.1, PDB: 5GJV). In conclusion, the M-motif is less defined by a specific amino acid sequence (Fig. 4B) but rather by its secondary structure and interactions.

There are two known groups of short loops/turns connecting secondary structure elements, which are both not defined by a characteristic secondary structure. The first is classified by its length and that the loops’ residues are not incorporated into the hydrogen bonding of the neighbouring secondary structure elements^46^. The other group is defined by the side chain (typically Asp, Asn, Ser or Thr) that interacts with the backbone but only moderately changes the backbone orientation and does not result in a pair of antiparallel helices^47^. The residues of the M-motif are all forming backbone hydrogen bonds with the neighbouring alpha-helices, which excludes the M-motif from the first group of turns. Frequently, there is an asparagine/serine in the M-motif that interacts with the C-terminal helix backbone, but in the M-motif, a complete turn is formed. Therefore, the M-motif does not strictly fit into any of the two known groups and defines its own category of inter-helix turns.

### Role of the SIL glutamate in Tspan17

For Tspan17, we find that mutating the SIL glutamate or the TMS1 N-terminal lysine reduces glycosylation and ER-exit (Fig. 5). In addition, the expression level is reduced, which could be a secondary effect of disturbed trafficking through the ER. Altogether, the three assays yield a consistent picture.

Importantly, glycosylation, ER-exit and expression are back to normal levels when the SIL glutamate and N-terminal lysine are swapped (Fig. 5). This points towards a functionally important glutamate-lysine interaction between the SIL and the TMS1 N-terminal lysine. Because the positions of the two oppositely charged amino acids can be interchanged, we speculate that the interaction is most likely of electrostatic nature.

In other tetraspanins, the SIL glutamate seems to be of relevance as well, although the overall picture is unclear. For instance, in CD9, glutamate/lysine mutations have no effect on ER exit (Fig. S12), but increase CD9 association with EWI-2 (Fig. S13). This is very interesting as it implies two things. First, without salt-bridge, CD9 still adopts a functional conformation, or in other words, lack of the salt-bridge does not lead to complete misfolding. Second, its higher affinity to EWI-2 may be explained by a switch towards an open conformation, as shown for CD81 that interacts in the open conformation stronger with its primary binding partner CD19^12^. In cryo-EM, CD9 interacts with EWI-2 not in a complete but partial open conformation^16^. The four helices still arrange in a funnel shape but the LEL is folded more upright. In complex with EWI-F, that is a EWI-2 homolog, cryo-EM reveals a CD9 conformation resembling the open conformation shown in Fig. 1B (see also reference^15^). Hence, elimination of the salt-bridge could trigger partial CD9 opening and enhance binding to EWI-2. Moreover, from the 33 human tetraspanins, we have performed mutational analysis of eight family members. In three cases each, mutation of the SIL glutamate either significantly decreases or increases expression (Figs. S8, S14).

Altogether, the picture is neither consistent nor complete and we are just at the beginning of understanding the mechanism by which the SIL modulates tetraspanin structure. In fact, we find it is not surprising that equivalent mutations produce different effects in different tetraspanins, as they have different binding partners and functions.

## Conclusion

In this study, we show that the SIL of tetraspanins contains a conserved five amino acid core sequence forming a structural motif that resembles the letter M. Using Tspan17 as example, we find that mutation of the SIL glutamate or the N-terminal lysine adjacent to TMS1 reduces glycosylation, ER-exit, and expression. All effects are back to normal levels upon position swapping of the two oppositely charged amino acids. We speculate that glutamate and lysine interact electrostatically, which might impact the tertiary structure and as a result modulate the interaction network of Tspan17.

## Supplementary Information

Supplementary Information.

## Data Availability

The datasets generated during and/or analysed during the current study are available from the corresponding author on reasonable request.
