# Supplementary material for: A conserved sequence in the small intracellular loop of tetraspanins forms an M-shaped inter-helix turn
Source: Sci Rep. 2022 Mar 16;12:4494. doi: 10.1038/s41598-022-07243-y (PMC8927573; doi:10.1038/s41598-022-07243-y)
Supplement: Supplementary file 1 — Supplementary Information. [file 41598_2022_7243_MOESM1_ESM.pdf]

Supplementary Data for

# A conserved sequence in the small intracellular loop of tetraspanins forms an M-shaped inter-helix turn

Nikolas Reppert[1,*] and Thorsten Lang[1,*]
[1]Department of Membrane Biochemistry, Life & Medical Sciences (LIMES) Institute, University of Bonn, Carl-Troll-Straße 31, 53115 Bonn, Germany

[*]Correspondence should be addressed to Nikolas Reppert (Nikolas.Reppert@uni-bonn.de) and Thorsten Lang (thorsten.lang@uni-bonn.de)

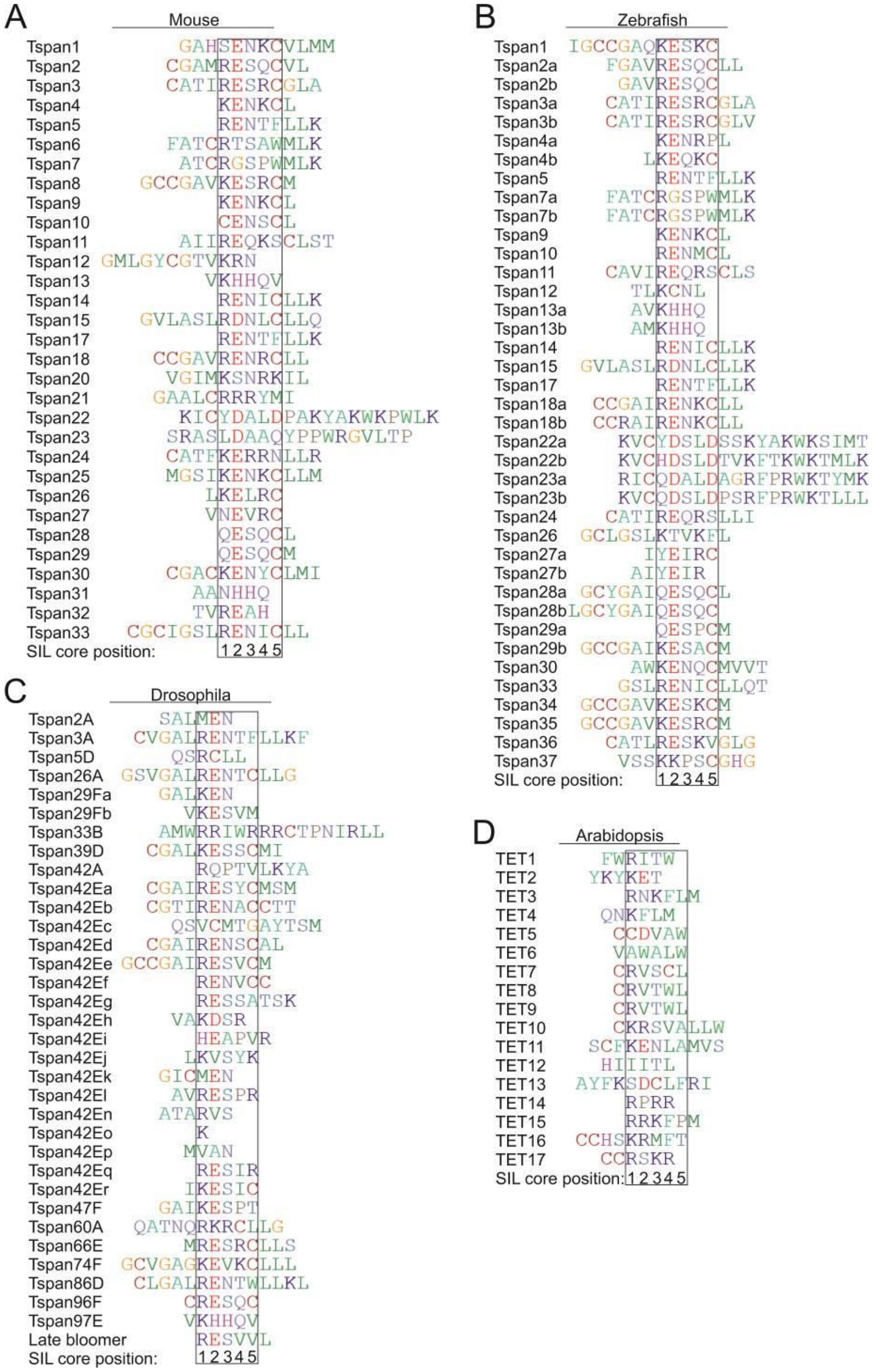


Figure S1: SIL core sequence alignments of other species.

Employing the same criteria as for human tetraspanins, SIL core sequences (boxes) are aligned from tetraspanins from (A) mouse (*Mus musculus*), (B) zebrafish (*Danio rerio*), (C) fruit fly (*Drosophila melanogaster*) and (D) *arabidopsis thaliana*. The alignment was created using BioEdit[1] v7.0.5 (http://www.mbio.ncsu.edu/BioEdit/bioedit.html).

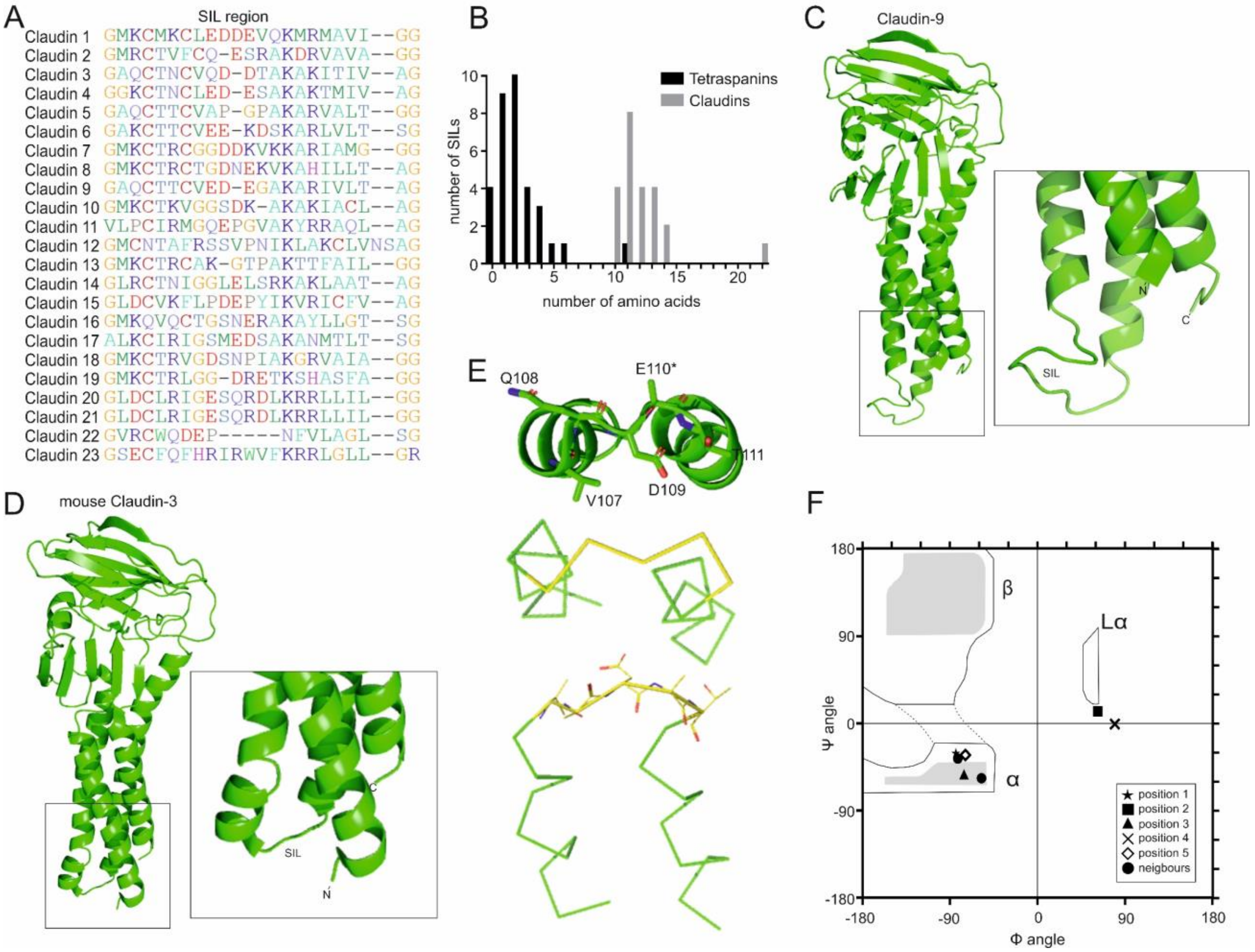


Figure S2: SIL in Claudins, a similarly structured protein family.
(A) ClustelW alignment of 23 human Claudins identified in the Uniprot database. SIL region between TMS2 and TMS3. The alignment was created using BioEdit[1] v7.0.5 (http://www.mbio.ncsu.edu/BioEdit/bioedit.html). (B) Distribution of the number of non-helical amino acids present between TMS2 and TMS3 (predicted by Jpred4) in human tetraspanins (black) and Claudins (grey). Please note that in some tetraspanins helical continuity between the two transmembrane segments is predicted, yielding a value of zero. In 19 Claudins, in the longer connecting segment a short beta-strand is predicted using Jpred4[2] (http://www.compbio.dundee.ac.uk/jpred/).(C) Crystal structure of human Claudin-9 (pdb: 6OV3) showing its SIL loop points away from the helix bundle. (D) Crystal structure of mouse Claudin-3 (pdb: 6AKF) with only a short SIL loop resembling an M-motif-like structure as seen for tetraspanins. From the boxed region, a magnified view is shown. (E) Different views from the Claudin-3 M-motif. Asterisk at E110 indicates that part of the side chain is unresolved in the crystal structure. (F) Ramachandran Plot of the Claudin-3 SIL. It does not fulfil the tetraspanin M-motif secondary structure criteria because position 3 is alpha-helical and position 4 is left handed helical. Therefore, we refer to this motif and more such examples (e.g. pdb: 4UJ6, AGGAK; pdb: 6I9D, DGSPD and pdb: 5WO6, YDNNE) as M-like-motifs. The data illustration was performed using GraphPad Prism version 6.04 for Windows (www.graphpad.com) and PyMOL 2.5 (https://pymol.org/2/).

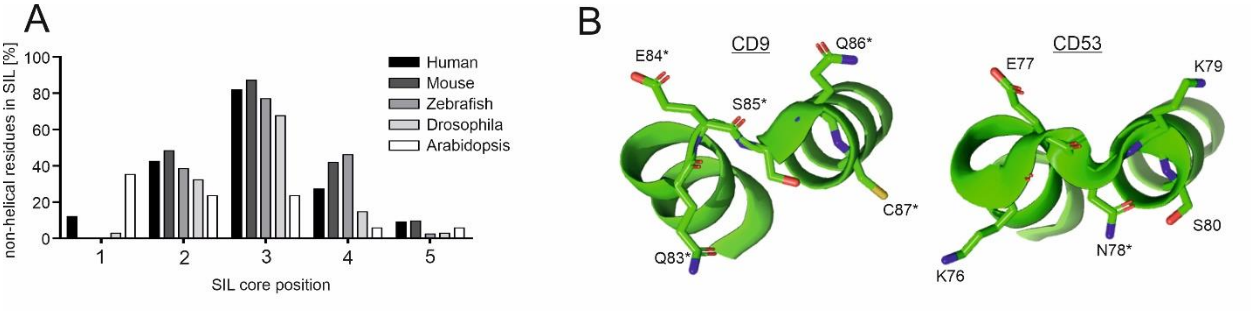


Figure S3: Non-helical structural elements in the SIL core.
(A) In different species (see legend), employing Jpred4 the percent of non-helical amino acids per SIL core position is predicted. In a minority of tetraspanins (Tspan2, 13, 28 and 32) the program suggests unrealistic continuous alpha-helicity between TMS2 and TMS3, that however is unrealistic due to the topology of the protein. For human Tspan22 the predicted helix gap was C-terminal of the SIL core sequence. The data illustration was performed using GraphPad Prism version 6.04 for Windows (www.graphpad.com). (B) Comparison of the theoretical average structure in (A) with crystallographic data of CD9 (pdb: 6K4J) and CD53 (pdb: 6WVG). CD9-S85 and CD53-N78 correspond to the third position of the SIL core. In line with the prediction, the amino acids at the SIL periphery are increasingly alpha helical. An asterisk indicates the amino acids that were predicted to be non-helical. The images were created using PyMOL 2.5 (https://pymol.org/2/).

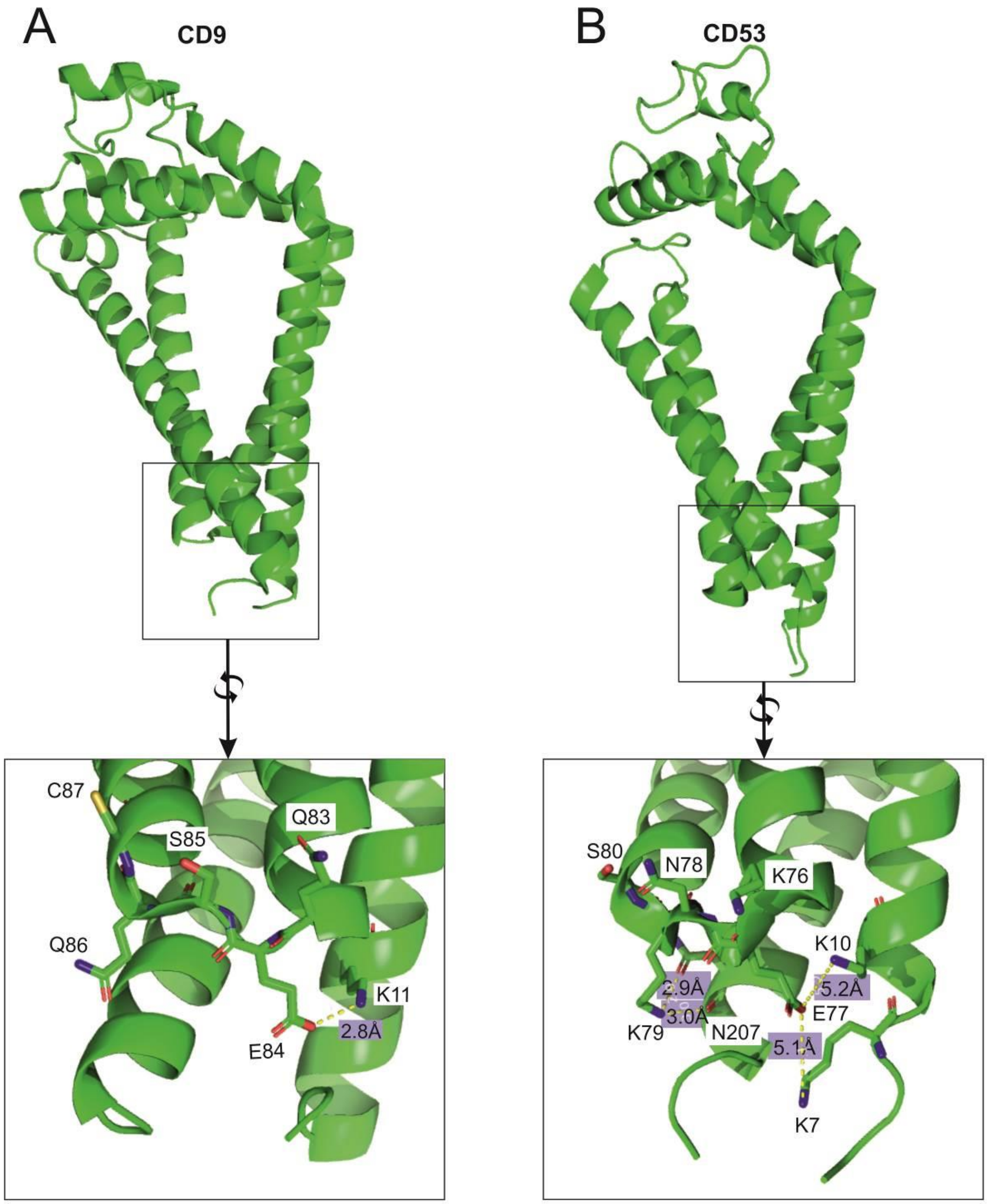


Figure S4: SIL-TMS interactions in CD9 and CD53.
Crystal structures of CD9 (pdb 6K4J; left) and CD53 (pdb 6WVG; right), illustrating the alpha-helical structure of the LELs (top) and the funnel-shaped arrangements of the TMSs (lower part). Large boxes; magnified views from the small boxed regions from a different perspective, such that top views of the M-motifs are visible, together with the N- and C-terminal segments. Amino acids of the SIL core regions (CD9, aa 83 – 87; CD53, 76 - 80), lysines of the N-terminal peptides (CD9, K11; CD53, K7, K11), and the asparagine (N207) within the C-terminal helix of CD53 are indicated. In CD9, a salt-bridge between E84 and K11 is illustrated by the dotted yellow line. The distance between the charged atoms is 2.8 Å (highlighted in purple). In CD53, distances between E77 and K7/K10 (yellow dotted lines) are too long (5.1Å/ 5.2Å, highlighted in purple) for a salt-bridge to form. The distances between the nitrogen in K79 and the carbonyl groups in N207 (side chain, 2.9Å; backbone, 3.0Å) allow for polar interactions (maximal interaction distance between C=O – H-N is 3.0Å[3]). The images were created using PyMOL 2.5 (https://pymol.org/2/).

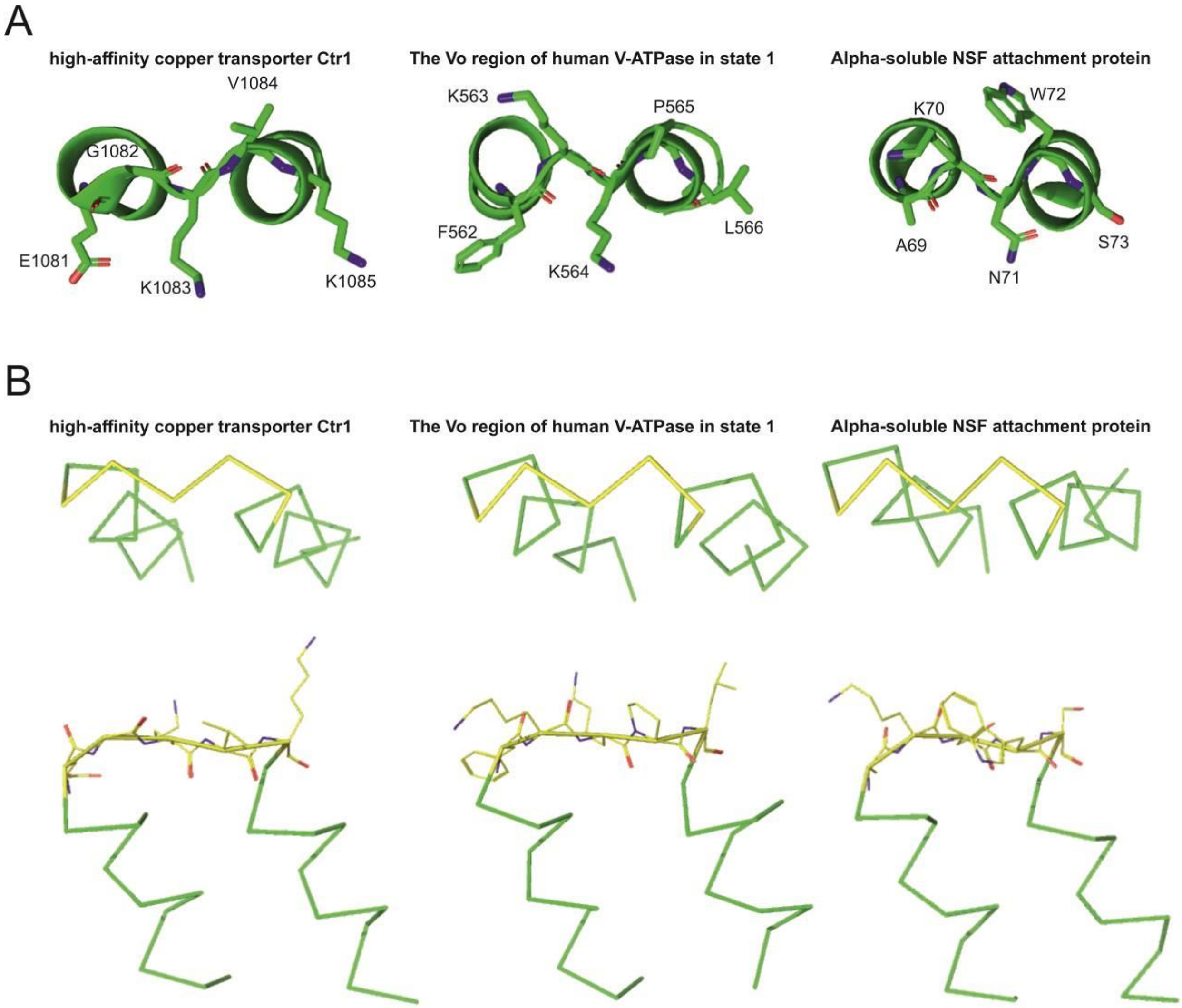


Figure S5: The M-motif in more non-tetraspanin proteins.
Different views from the M-motifs of the high-affinity copper transporter Ctr1 (pdb: 6M98), V-ATPase (pdb: 6WLW) and alpha-soluble NSF attachment protein (pdb: 6IP1). (A) Cartoon and (B) ribbon diagrams with the amino acids of the SIL core shown in detail as yellow sticks (the naming of the modes of illustration refer to PyMOL). Side chain colour code is red for oxygen and blue for nitrogen. Residue numbering in (A) refers to the protein sequence and may differ from the sequence number in the crystal structures. For M-motifs in Claudin-3 see Fig. S2E. Images were created using PyMOL 2.5 (https://pymol.org/2/).

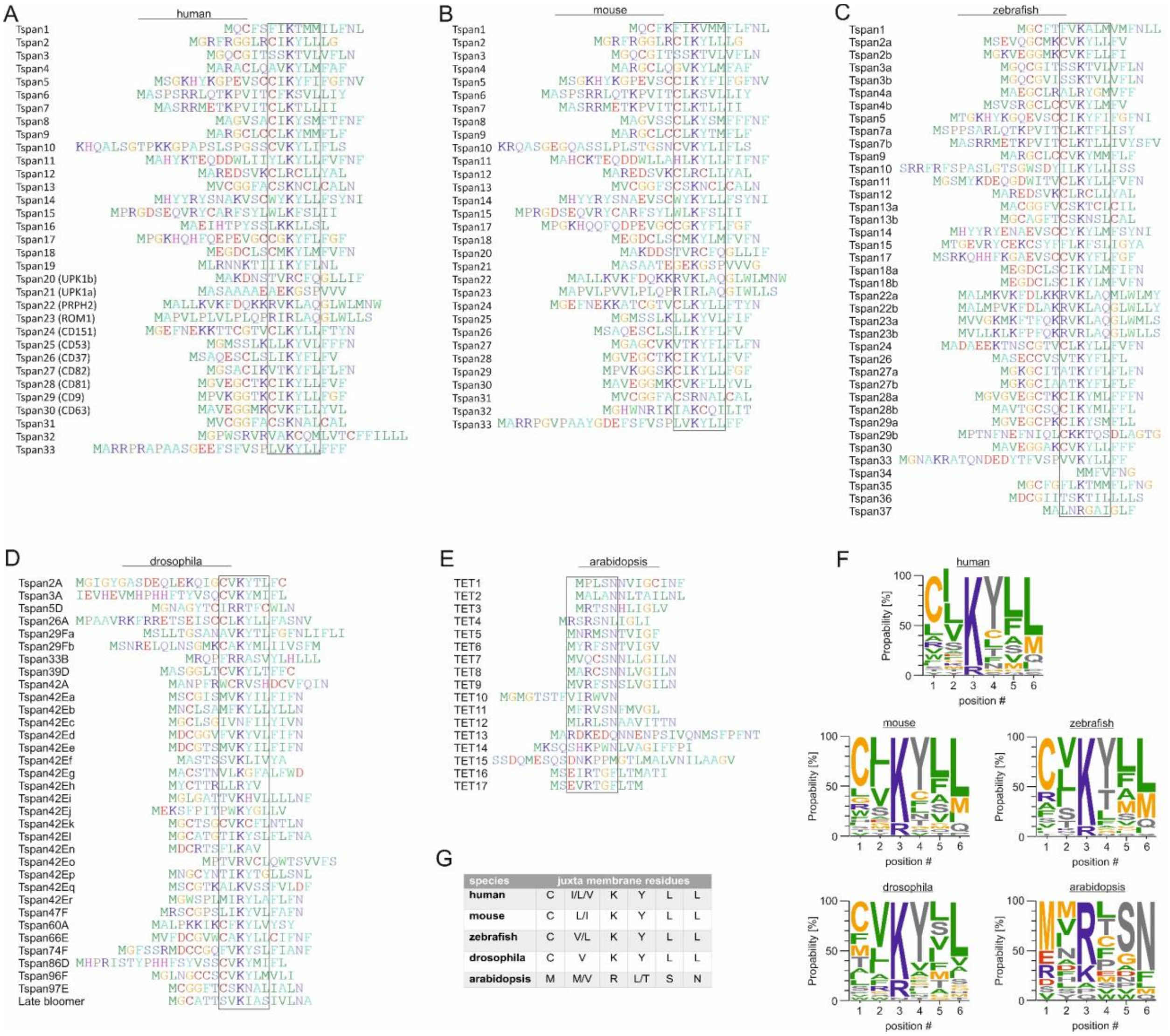


Figure S6: Alignment of the N-terminal tetraspanin sequences from different species.

(A - E) N-terminal sequences plus the first five amino acids of TMS1 are aligned using tetraspanin sequences of (A) human (*Homo sapiens*), (B) mouse (*Mus musculus*), (C) zebrafish (*Danio rerio*), (D) fruit fly (*Drosophila melanogaster*) and (E) arabidopsis (*Arabidopsis thaliana*). The sequences were aligned with reference to the most C-terminal positively charged amino acid of the N-terminus, yielding in the juxta membrane region a conserved sequence of six amino acids including the lysine/arginine used for alignment (see boxes). (F) Residue probability of the six amino acids based on (A - E). The sequence is similarly conserved in animal tetraspanins but different in arabidopsis. (G) Consensus sequences in the juxtamembrane regions defined as described in the legend of Figure 2. The alignment was created using BioEdit[1] v7.0.5 (http://www.mbio.ncsu.edu/BioEdit/bioedit.html) and the illustration of residue frequency was done using Weblogo[4] 3.7.4 (https://weblogo.berkeley.edu/logo.cgi).

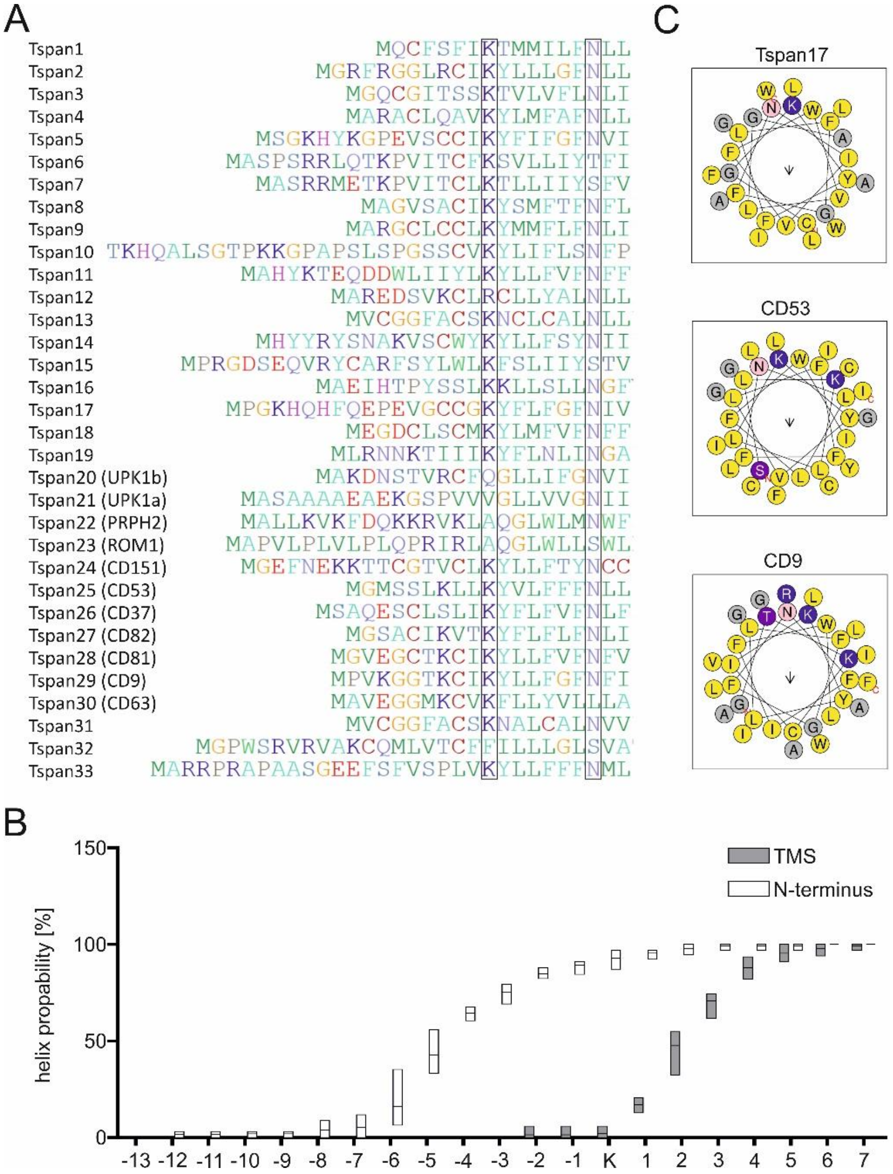


Figure S7: Orientation of the conserved juxtamembrane lysine in relation to the conserved asparagine in TMS1.
(A) Alignment of the human tetraspanin juxtamembrane/TMS1 starting sequences with reference to a conserved asparagine (right box). The left box marks the amino acid seven positions upstream, which frequently is a lysine (see also Fig S6). The long N-terminus of Tspan10 is deleted for this illustration. Seven positions distance roughly equal two turns of an alpha helix (one alpha helical turn corresponds to 3.6 residues[5]) and as a result a similar orientation of the residues. (B) To test whether the conserved lysine and asparagine are located to the same alpha-helix the structure of each tetraspanins' juxtamembrane region (s. Fig S6) was predicted employing Jpred4[2] (http://www.compbio.dundee.ac.uk/jpred/) or TMHMM[6,7] Server v. 2.0 (http://www.cbs.dtu.dk/services/TMHMM/). The results whether an amino acid of a certain tetraspanin is within an alpha-helix and/or within a TMS were averaged per species and illustrated in a box plot (highest and lowest values framing the box, line in the middle is the mean value for all species; conserved lysine was named K, residues N-terminal of the lysine are named -13 to -1 and C-terminal 1 to 7). This illustration shows that this particular conserved lysine is located to an alpha-helix (in 93% of analysed tetraspanins). The predicted TMS1 probability increases directly C-terminal of the lysine. The lysine is not located to the TMS1 but to the N-terminal helix extension of it. Therefore, it is located to the same helix as the conserved asparagine. (C) The wheel plots of the juxtamembrane/TMS1 sequences of Tspan17, CD53 and CD9 show again the similar orientation of the lysine and asparagine. The wheel plots analyse the amphipathic character of the helix, yielding its hydrophobic moment, which is indicated by the orientation and length of the arrow in the center of the helix. The wheel plots were created using HeliQuest[8] (https://heliquest.ipmc.cnrs.fr/cgi-bin/ComputParams.py).

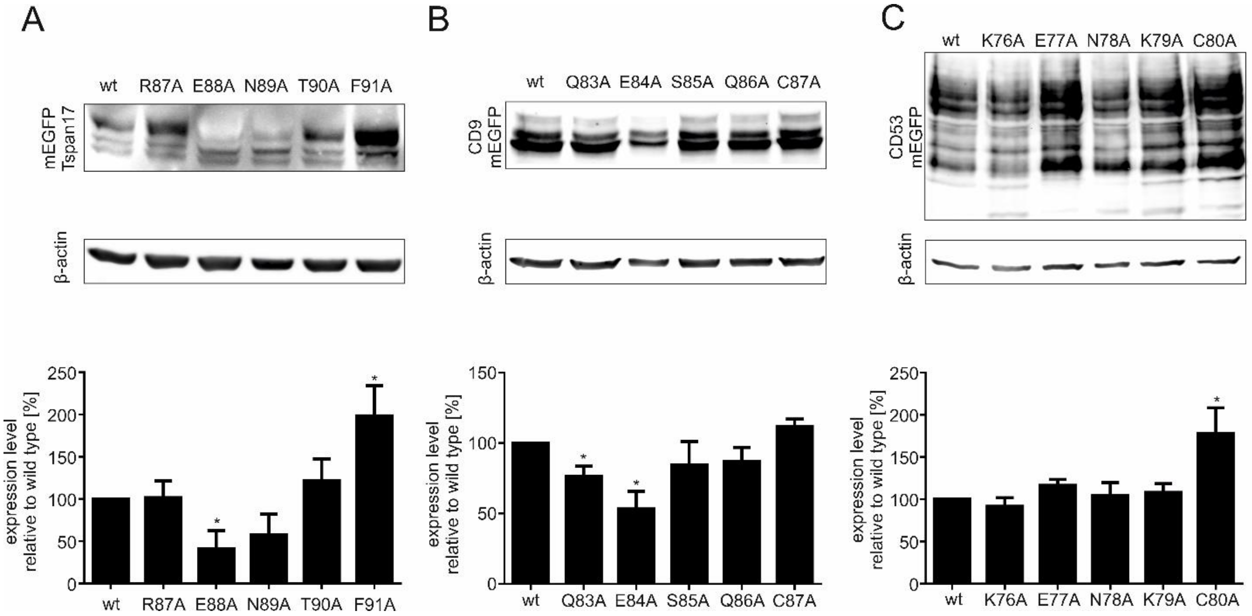


Figure S8: Mutations in the SIL consensus region occasionally change the expression level.
In GFP-tagged (A) Tspan17, (B) CD9 and (C) CD53, single SIL core amino acids are exchanged to alanine. Constructs are expressed in HepG2 cells, and expression levels are analysed by Western Blot. Upper panels, representative Western Blot membranes. Please note that upon mutation of the second and third position in Tspan17 the higher running GFP-Tspan17 band vanishes (A), which is due to a lack in glycosylation (see also Fig. S9). Lower panels, quantification of the Western blot bands. GFP signal is related to actin used as loading control, and normalized to wild-type (set to 100%). Values are given as means ± SD (n = 4). The statistical analysis was done employing a repeated measures ANOVA comparing each mutation to the wild-type (*P < 0.05, **P < 0.01, ***P < 0.001, ****P < 0.0001). The full blots are shown in the supplementary data (Fig. S17-S19). The data analysis and illustration was performed using Fiji-ImageJ[9] (https://imagej.net/) and GraphPad Prism version 6.04 for Windows (www.graphpad.com), respectively.

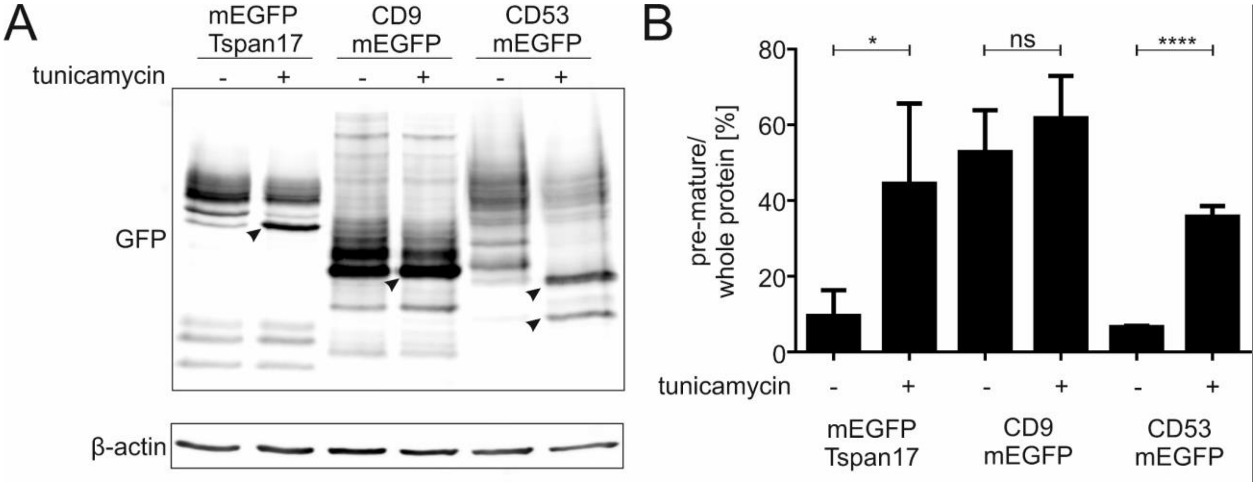


Figure S9: Band pattern of Tspan17, CD9 and CD53 without and with inhibition of glycosylation by tunicamycin. (A) HepG2 cells are transfected with mEGFP-Tspan17, CD9-mEGFP or CD53-mEGFP and 5 µg/ml tunicamycin is added to the medium. Tunicamycin inhibits N-glycosylation; there are three potential N-glycosylation sites in Tspan17, two in CD53 and two in CD9 (predicted by NetNGlyc[10] 1.0; http://www.cbs.dtu.dk/services/NetNGlyc/). After 18h of incubation, cells are lysed and analysed via Western blot. In the presence of tunicamycin, some bands increase in intensity. The ones with the highest increase were highlighted (see arrowheads) and assumed to represent the pre-mature protein. (B) The diminishment of protein maturation by tunicamycin is quantified by relating the amount of pre-mature to whole protein. There is a significant increase in pre-mature protein in Tspan17 and CD53 but not in CD9. Still, we observe at least two prominent CD9 bands that could reflect a palmitoylated- and non-palmitoylated state, as also non-glycosylated CD81[11] shows up in the palmitoylated- and non-palmitoylated state as two clearly separated bands[12]. CD9 maybe not glycosylated because both overlapping glycosylation sites are located in the small extracellular loop, that could be shielded by the large extracellular loop[13]. The result is used in Fig. 5 to assign the western blot bands to the mature and pre-mature forms of Tspan17. Values are given as means ± SD (n = 4). For statistics a two-tailed paired t-test is used (*P < 0.05, **P < 0.01, ***P < 0.001, ****P < 0.0001). Blots used in this analysis are shown in Fig. S25. The data analysis and illustration was performed using Fiji-ImageJ[9] (www. https://imagej.net/) and GraphPad Prism version 6.04 for Windows (www.graphpad.com), respectively.

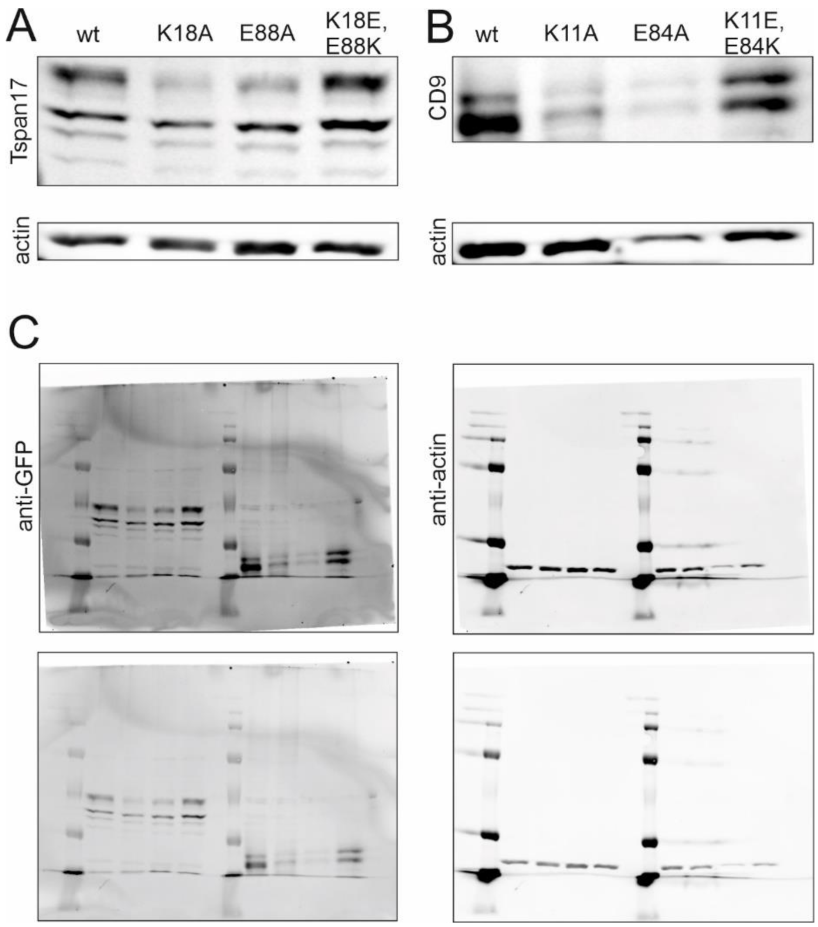


Figure S10: Expression of Tspan17 and CD9 glutamate-/lysine-mutations shortly after transient transfection
The expression of (A) mEGFP-Tspan17 constructs and (B) CD9-mEGFP constructs as indicated 5h after transient transfection (which we identified as the onset of expression) visualized by Western blot analysis. The upper panel shows the GFP signal and the lower one the actin signal. Compared to wild-type tetraspanins, though the respective actin bands are similar to control, the Tspan17-K18 and CD9-K11A bands are weaker, indicating lower expression levels already 5 h after transfection. In case of CD9, this is perhaps due to toxicity of the constructs (see Fig. S15) and not due to a maturation or trafficking defect. (C) Whole blots are shown in two different scalings. The data illustration was performed using Fiji-ImageJ[4] (https://imagej.net/).

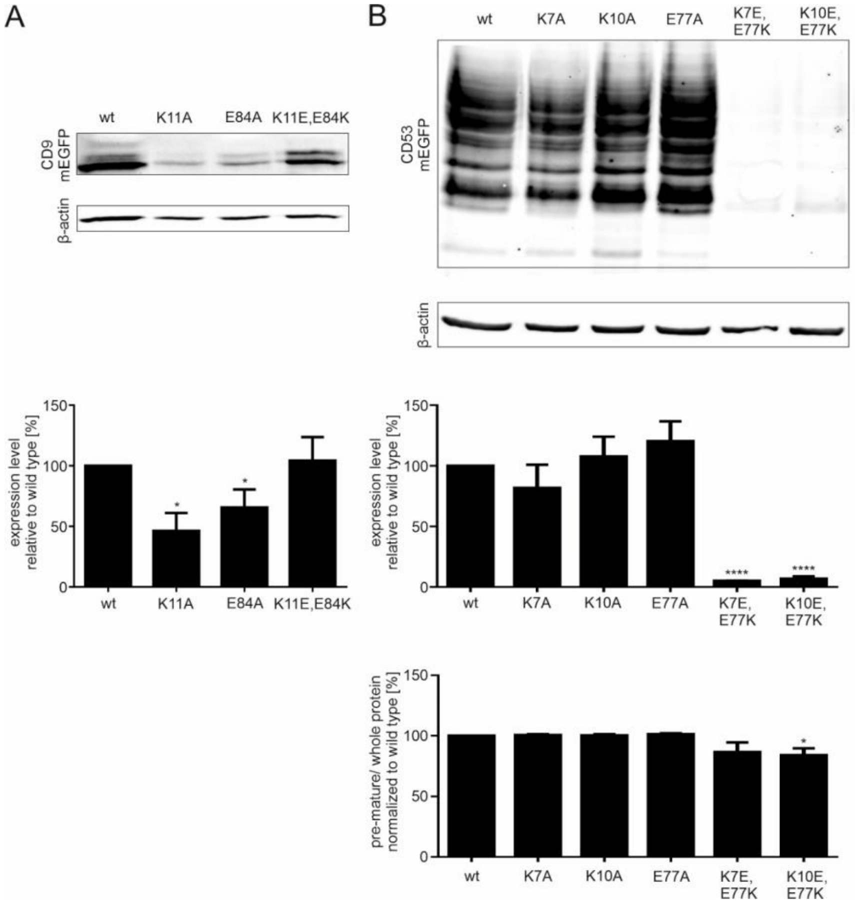


Figure S11: Expression level after disrupting/rescuing the glutamate-lysine interaction in CD9 and CD53.
In GFP-tagged (A) CD9 and (B) CD53, glutamate or lysine are exchanged to alanine, or their positions are exchanged (respective mutants in Tspan17 are analysed in Fig. 5). Constructs are expressed in HepG2 cells, and expression levels are quantified by Western blot. Upper panels show representative Western blot membranes. Below, quantification of the expression levels. To this end, the GFP band intensities are related to the actin band intensity (to correct for different loading). Values are then further related to wild-type (set to 100%). The strong reduction in expression of the CD53 double mutants is unexpected but could be explained by the change in charge distribution caused by the glutamate/lysine swap. The negative effect of altered charges in the juxtamembrane region could be strong in a tetraspanin without a stabilizing salt-bridge (please note that in the crystal structure of CD53 the distance between the two amino acids actually is too long for a salt-bridge to form). Lower right panel; for CD53, additionally the pre-mature protein is related to whole protein to test for any defects in glycosylation (see Fig. S9). Values are given as means ± SD (n = 4). The statistical analysis was done employing a repeated measures ANOVA comparing each mutation with the wild type (*P < 0.05, **P < 0.01, ***P < 0.001, ****P < 0.0001). The full blots are shown in Fig. S21 and S22. The data analysis and illustration was performed using Fiji-ImageJ[9] (www. https://imagej.net/) and GraphPad Prism version 6.04 for Windows (www.graphpad.com), respectively. For a diminishment in Tspan17 and CD9 expression shortly after transfection see Fig. S10. CD9 mutants seems to have a cytotoxic effect (Fig. S15).

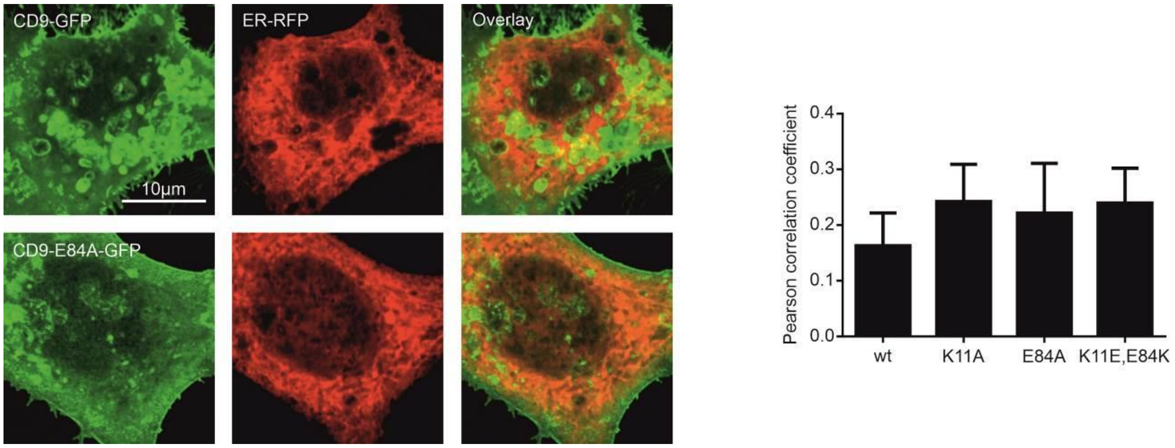


Figure S12: Co-localization of the ER with CD9 or CD9 constructs in which the salt-bridge is disrupted, or positions of the charged amino acids are interchanged

The co-localization of the ER with CD9-mEGFP and salt-bridge mutants was analyzed recording confocal images of the GFP and the RFP channel, the latter showing RFP fused to an ER marker. For quantification, the Pearson correlation coefficient was calculated. Values are given as means ± SD (n = 3; for each biological replicate 10 cells were imaged). The statistical analysis was done employing a repeated measures ANOVA comparing each mutation with the wild type (*P < 0.05, **P < 0.01, ***P < 0.001, ****P < 0.0001). The data analysis and illustration was performed using Fiji-ImageJ[4] (www. https://imagej.net/) and GraphPad Prism version 6.04 for Windows (www.graphpad.com), respectively.

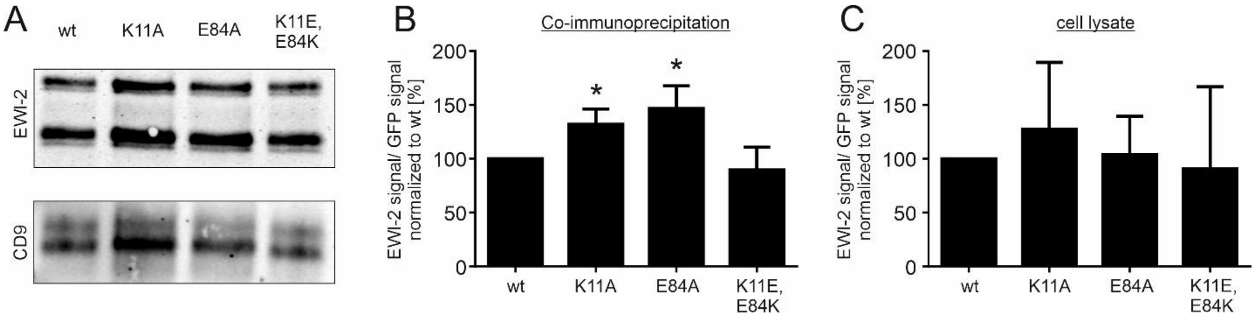


Figure S13: Co-Immunoprecipitation of EWI-2 with CD9 or CD9 mutants.
HepG2 cells express myc-tagged EWI-2 together with GFP labelled CD9 wild-type, CD9 mutants in which the salt-bridge is disrupted (K11A and E84A), or a mutant in which charges are swapped (K11E, E84K). Using an antibody that recognizes the LEL only if disulfide bridges are formed, we tested whether the disulfide-bridge forms in the mutants[14]. Albeit less efficient, all mutants were readily recognized by the antibody (see Fig. S16). For maintaining the tetraspanin-interaction network, cells were lysed with 1% CHAPS, followed by immunoprecipitation of GFP using GFP-trap beads. (A) Samples are analysed by Western blot, staining membranes for EWI-2 (top; please note that two bands are visible, one for EWI-2 and EWI-2-Wint each, both included in the quantification) and GFP (bottom; visualizing the GFP-labelled CD9 constructs as indicated). (B) Quantification of the precipitate. The ratio between the EWI-2 and GFP band intensities, normalized to wild-type (set to 100%), is used as a measure for EWI-2 pull down efficiency. Values are given as means ± SD (n = 4). (C) Relative expression levels of EWI-2 and GFP-labelled CD9 and CD9 mutants in the cell lysate. Although expression levels are highly variable, the ratio between EWI-2 and the GFP-construct is similar in the different immunoprecipitation experiments. Please note that blot #1 shown in Fig. S.26 could not be included in (C) due to low signal in the lysate. Values are given as means ± SD (n = 3). For statistical analysis, we employed a repeated measures ANOVA test comparing the mutations to the wild type (*P < 0.05, **P < 0.01, ***P < 0.001, ****P < 0.0001). Full blots are shown in Fig. S26. The data analysis and illustration was performed using Fiji-ImageJ[9] (www. https://imagej.net/) and GraphPad Prism version 6.04 for Windows (www.graphpad.com).

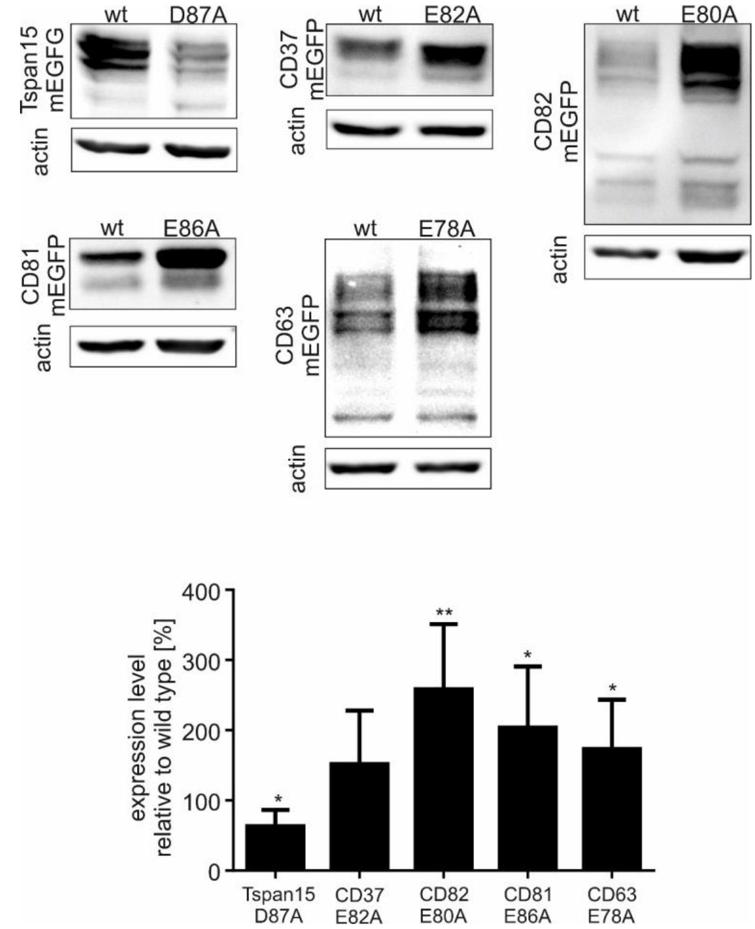


Figure S14: Mutation of SIL position 2 in Tspan15, CD37, CD82, CD81 and CD63
Same experiment as in Fig. S8, mutating only glutamate/aspartate at position 2 to alanine in Tspan15 (D87A), CD37 (E82A), CD82 (E80A), CD81 (E86A) and CD63 (E78A). Expression levels are normalized to actin and related to the respective wild-type protein (set to 100%). Values are given as means ± SD (n = 6 - 7). For statistical analysis a two-tailed paired t-test was used (*P < 0.05, **P < 0.01, ***P < 0.001, ****P < 0.0001). Full blots are shown in Fig. S23 and S24. The data analysis and illustration was performed using Fiji-ImageJ[9] (https://imagej.net/) and GraphPad Prism version 6.04 for Windows (www.graphpad.com), respectively.

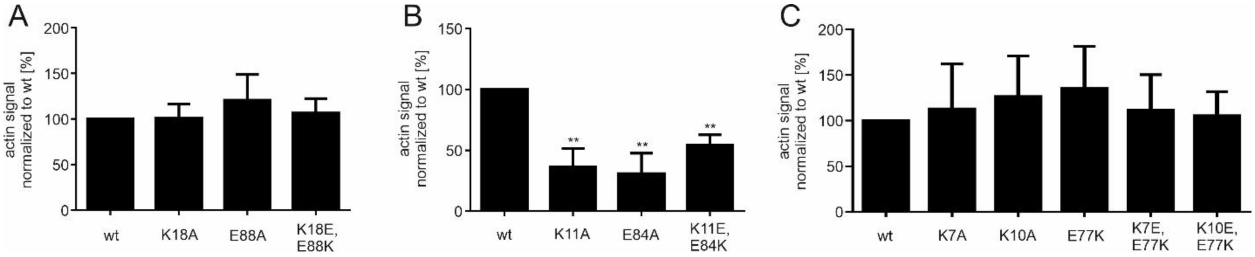


Figure S15. Cell toxicity of some mutants

Expression of (A) Tspan17, (B) CD9 or (C) CD53 or the mutants as indicated. Same experiments as in Figs. 5 and S11, showing quantification of the actin band intensities. In these experiments, a defined amount of HepG2 cells (1.8 Mio) is transfected with the GFP-labelled constructs. Under the assumption that actin levels are proportional to cell survival, we observe toxicity of the CD9 mutants. Should cytotoxicity preferentially affect strongly expressing cells, we would overestimate the reduction in expression in Fig. S11A. The statistical analysis was done by a repeated measures ANOVA test comparing the mutations to the respective wild-type protein (*$P < 0.05$, **$P < 0.01$, ***$P < 0.001$, ****$P < 0.0001$). Values are given as means ± SD (n = 4). The data analysis and illustration is performed using Fiji-ImageJ[4] (www. https://imagej.net/) and GraphPad Prism version 6.04 for Windows (www.graphpad.com), respectively.

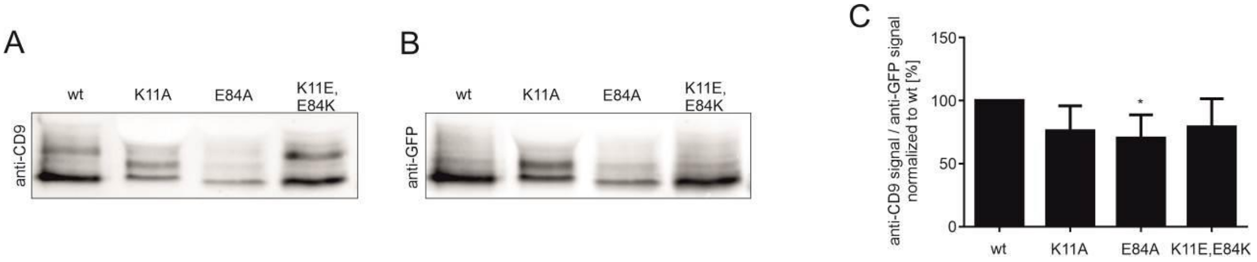


Figure S16: LEL disulfide-bond formation in CD9 mutants

HepG2 cells expressing GFP-labelled CD9 wild type or mutants are subjected to SDS-PAGE Western blot analysis under non-reducing conditions. Membranes are double stained with (A) a CD9 antibody recognizing the LEL only if a disulfide bond has formed[14] and (B) a GFP antibody. A decrease in the CD9-LEL signal relative to the GFP signal indicates absence/diminishment of the disulfide bond. The complete GFP and CD9-LEL signal (as seen in (A) and (B)) was used for this analysis. The CD9 wild type:GFP ratio is used as reference and set to 100%. Values are given as means $\pm$ SD (n = 6). Please note that a fraction of the K11A mutation runs at higher molecular weight as wild type, which however, is not reproducible (Fig. S27). Full blots are shown in Fig. S27. The data analysis and illustration was performed using Fiji-ImageJ[4] (www. https://imagej.net/) and GraphPad Prism version 6.04 for Windows (www.graphpad.com), respectively.

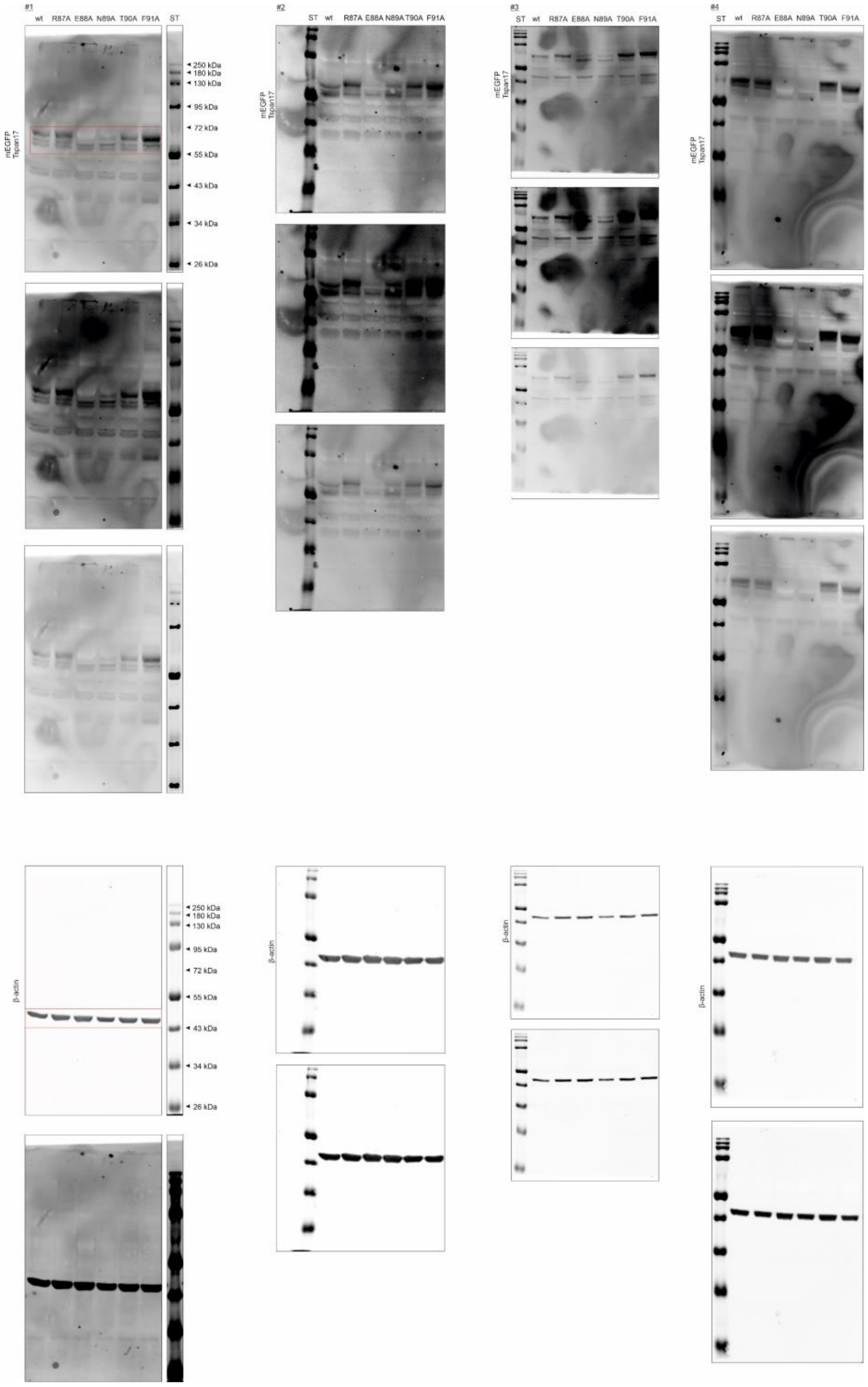


Figure S17: Full-length blots used for Figure S8.
The four blots (#1-4) for mEGFP-Tspan17 SIL core sequence mutations are depicted stained for GFP (upper panel) and for actin (lower panel). They represent the four blots used in the analysis in figure S8 (n=4). They are shown with protein standard (ST, 10-250 kDa range; NEB, #P7719S) and with three (GFP) or two (actin) different scalings to ensure clear visibility of all bands. The cutouts shown in figure S8 are highlighted by a red box in blot #1. During blot imaging the focus lay on the area framed by the protein standard, therefore all blots used for this analysis (Fig. S8) are shown. The data illustration was performed using Fiji-ImageJ[9] (https://imagej.net/).

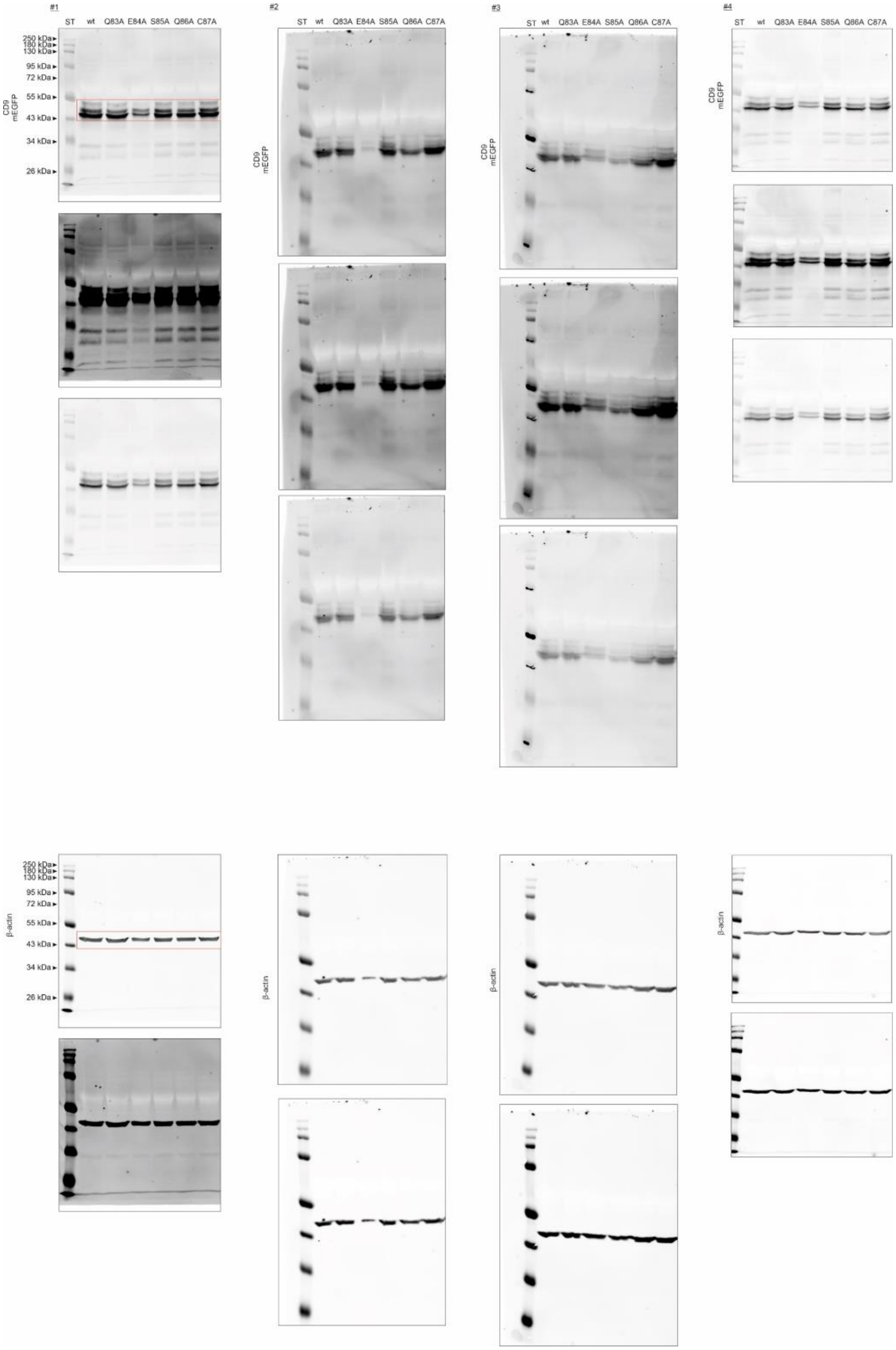


Figure S18: Full-length blots used for Figure S8.
The four blots (#1-4) for CD9-mEGFP SIL core sequence mutations are depicted stained for GFP (upper panel) and for actin (lower panel). They represent the four blots used in the analysis in figure S8 (n=4). They are shown with protein standard (ST, 10-250 kDa range; NEB, #P7719S) and with three (GFP) or two (actin) different scalings to ensure clear visibility of all bands. The cutouts shown in figure S8 are highlighted by a red box in blot #1. During blot imaging the focus lay on the area framed by the protein standard, therefore all blots used for this analysis (Fig. S8) are shown. The data illustration was performed using Fiji-ImageJ[9] (https://imagej.net/).

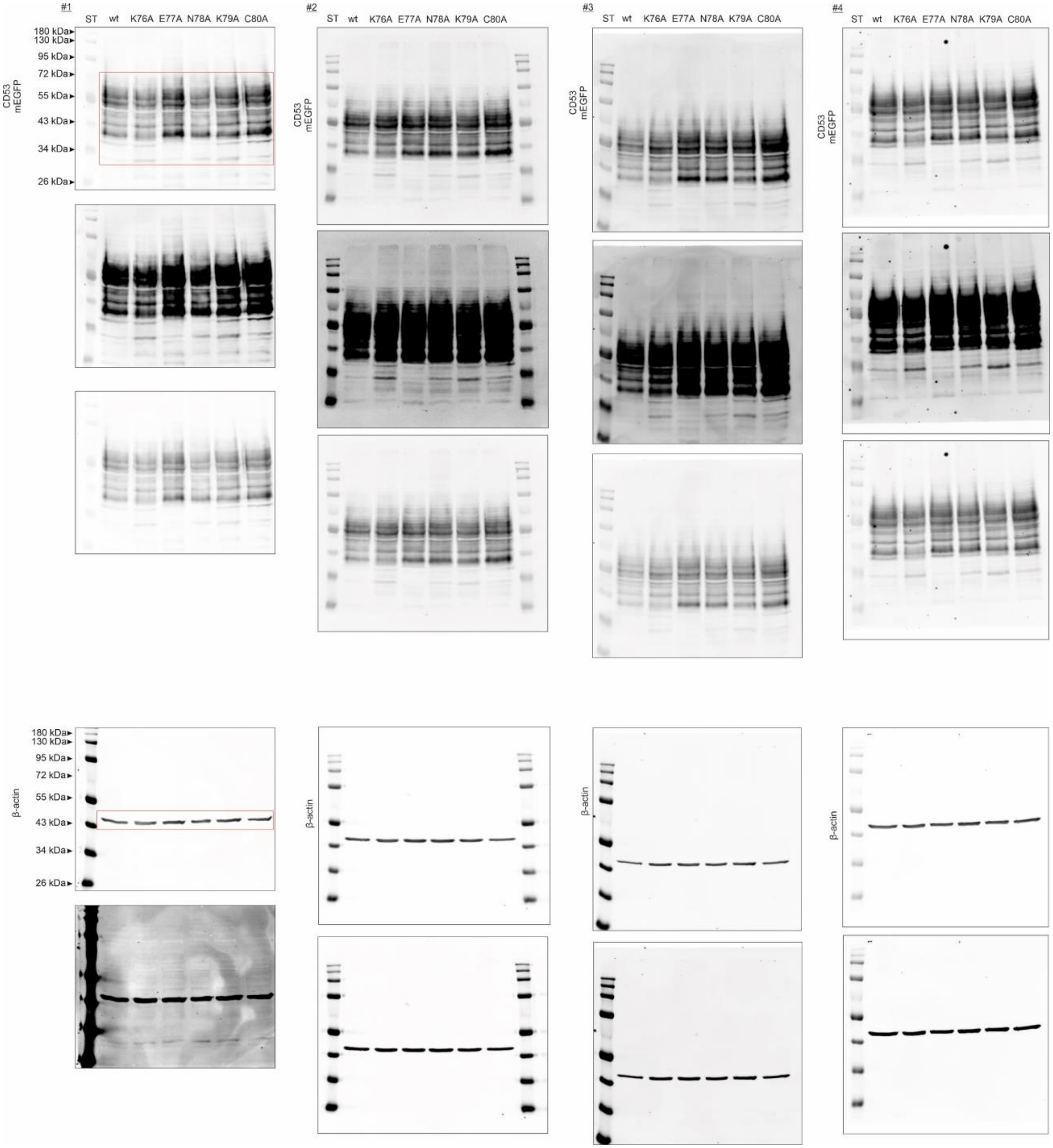


Figure S19: Full-length blots used for Figure S8.
The four blots (#1-4) for CD53-mEGFP SIL core sequence mutations are depicted stained for GFP (upper panel) and for actin (lower panel). They represent the four blots used in the analysis in figure S8 (n=4). They are shown with protein standard (ST, 10-250 kDa range; NEB, #P7719S) and with three (GFP) or two (actin) different scalings to ensure clear visibility of all bands. The cutouts shown in figure S8 are highlighted by a red box in blot #1. During blot imaging the focus lay on the area framed by the protein standard, therefore all blots used for this analysis (Fig. S8) are shown. The data illustration was performed using Fiji-ImageJ[9] (https://imagej.net/).

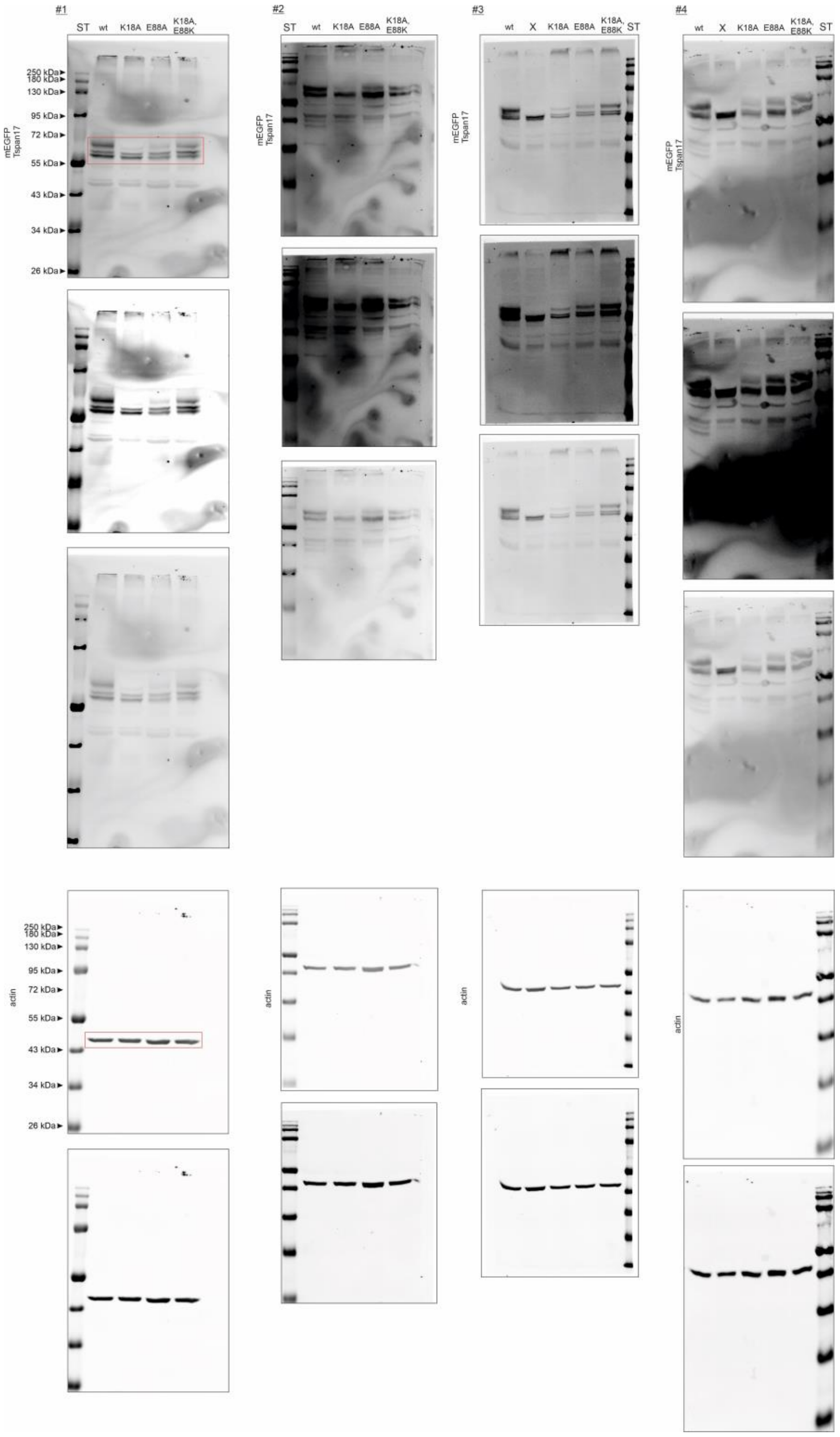


Figure S20: Full-length blots used in Figure 5
The four blots (#1-4) for mEGFP-Tspan17 salt-bridge mutations and rescue mutation are depicted stained for GFP (upper panel) and for actin (lower panel). They represent the four blots used in the analysis in figure 5 (n=4). They are shown with protein standard (ST, 10-250 kDa range; NEB, #P7719S) and with three (GFP) or two (actin) different scalings to ensure clear visibility of all bands. The cutouts shown in figure 5 are highlighted by a red box in blot #1. During blot imaging the focus lay on the area framed by the protein standard, therefore all blots used for this analysis (Fig. 5) are shown. The data illustration was performed using Fiji-ImageJ[9] (https://imagej.net/).

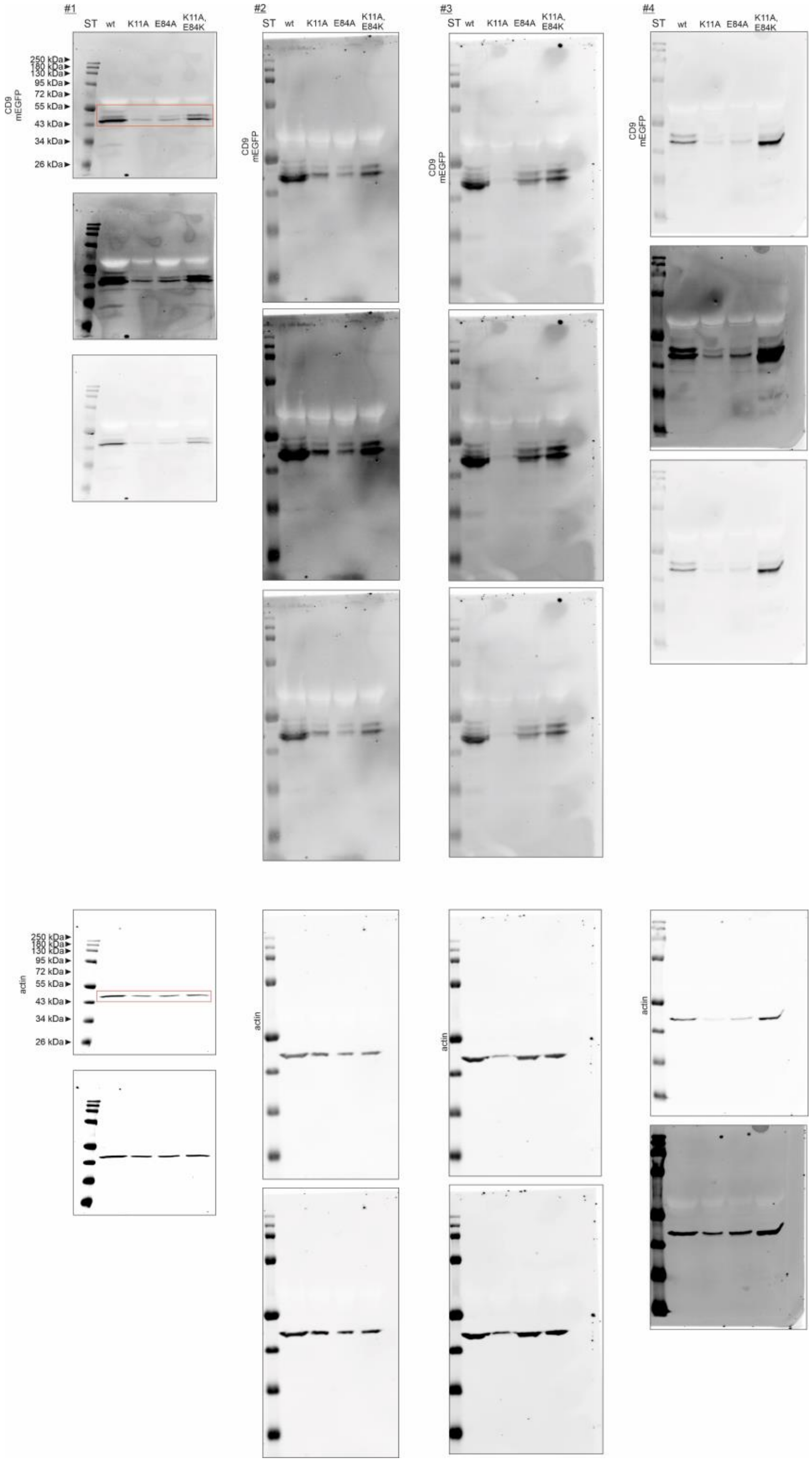


Figure S21: Full-length blots used in Figure S11
The four blots (#1-4) for CD9-mEGFP salt-bridge mutations and rescue mutation are depicted stained for GFP (upper panel) and for actin (lower panel). They represent the four blots used in the analysis in figure S11 (n=4). They are shown with protein standard (ST, 10-250 kDa range; NEB, #P7719S) and with three (GFP) or two (actin) different scalings to ensure clear visibility of all bands. The cutouts shown in figure S11A are highlighted by a red box in blot #1. During blot imaging the focus lay on the area framed by the protein standard, therefore all blots used for this analysis (Fig. S11) are shown. The data illustration was performed using Fiji-ImageJ[4] (https://imagej.net/).

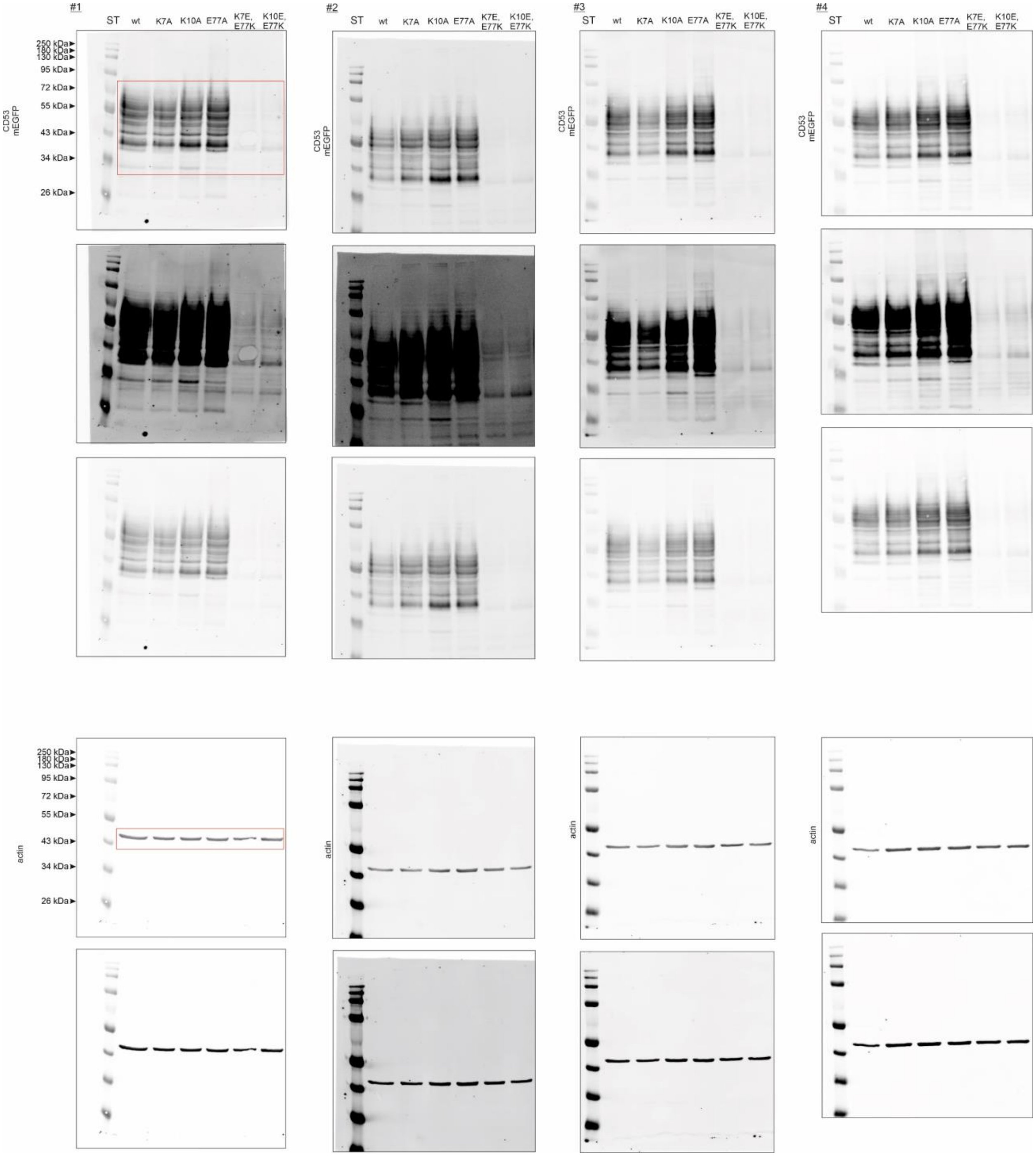


Figure S22: Full-length blots used in Figure S11

The four blots (#1-4) for CD53-mEGFP salt-bridge mutations and rescue mutation are depicted stained for GFP (upper panel) and for actin (lower panel). They represent the four blots used in the analysis in figure S11 (n=4). They are shown with protein standard (ST, 10-250 kDa range; NEB, #P7719S) and with three (GFP) or two (actin) different scalings to ensure clear visibility of all bands. The cutouts shown in figure S11B are highlighted by a red box in blot #1. During blot imaging the focus lay on the area framed by the protein standard, therefore all blots used for this analysis (Fig. S11) are shown. The data illustration was performed using Fiji-ImageJ[4] (https://imagej.net/).

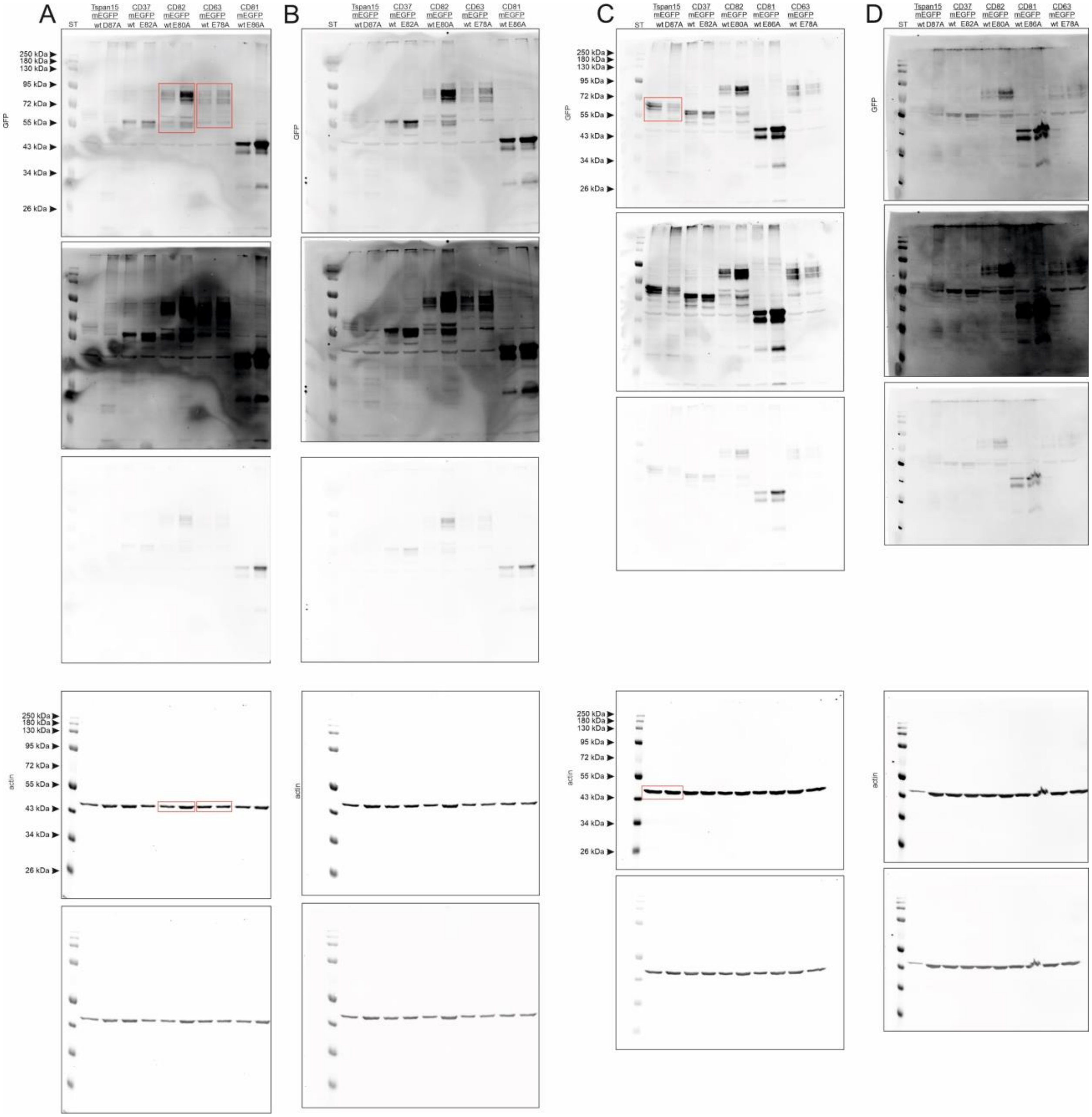

Figure S23: Full-length blots used in Figure S14.
The blots for figure S14 were divided onto two figures (Fig. S23 and S24). The seven blots (A-G, for E-G see Fig S24) for Tspan15, CD37, CD82, CD81 and CD63 are depicted stained for GFP (upper panel) and for actin (lower panel). They are shown with protein standard (ST, 10-250 kDa range; NEB, #P7719S) and with three (GFP) or two (actin) different scalings to ensure clear visibility of all bands. The cutouts used in figure S14 are highlighted by a red box. During blot imaging the focus lay on the area framed by the protein standard, therefore all blots used for this analysis (Fig. S14) are shown. The data illustration was performed using Fiji-ImageJ[9] (https://imagej.net/).

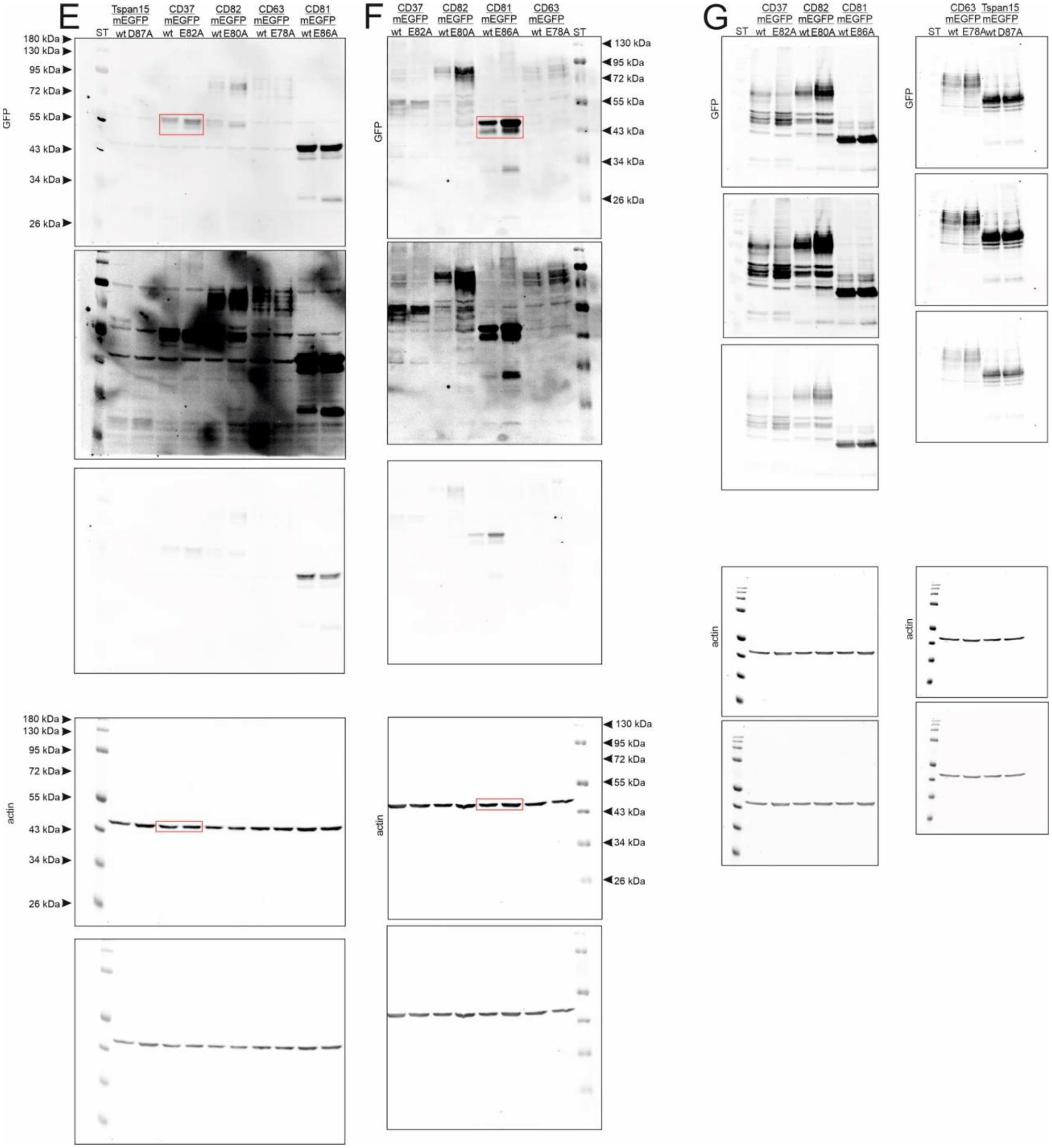


Figure S24: Full-length blots used in Figure S14.

The blots for figure S14 were divided onto two figures (Fig. S23 and S24). The seven blots (A-G, For A-D see Fig S23) for Tspan15, CD37, CD82, CD81 and CD63 are depicted stained for GFP (upper panel) and for actin (lower panel). They are shown with protein standard (ST, 10-250 kDa range; NEB, #P7719S) and with three (GFP) or two (actin) different scalings to ensure clear visibility of all bands. For Tspan15 there were only 6 samples analysed (n=6) which is the reason Tspan15 is missing on blot (F). The cutouts used in figure S14 are highlighted by a red box. During blot imaging the focus lay on the area framed by the protein standard, therefore all blots used for this analysis (Fig. S14) are shown. The data illustration was performed using Fiji-ImageJ[9] (https://imagej.net/).

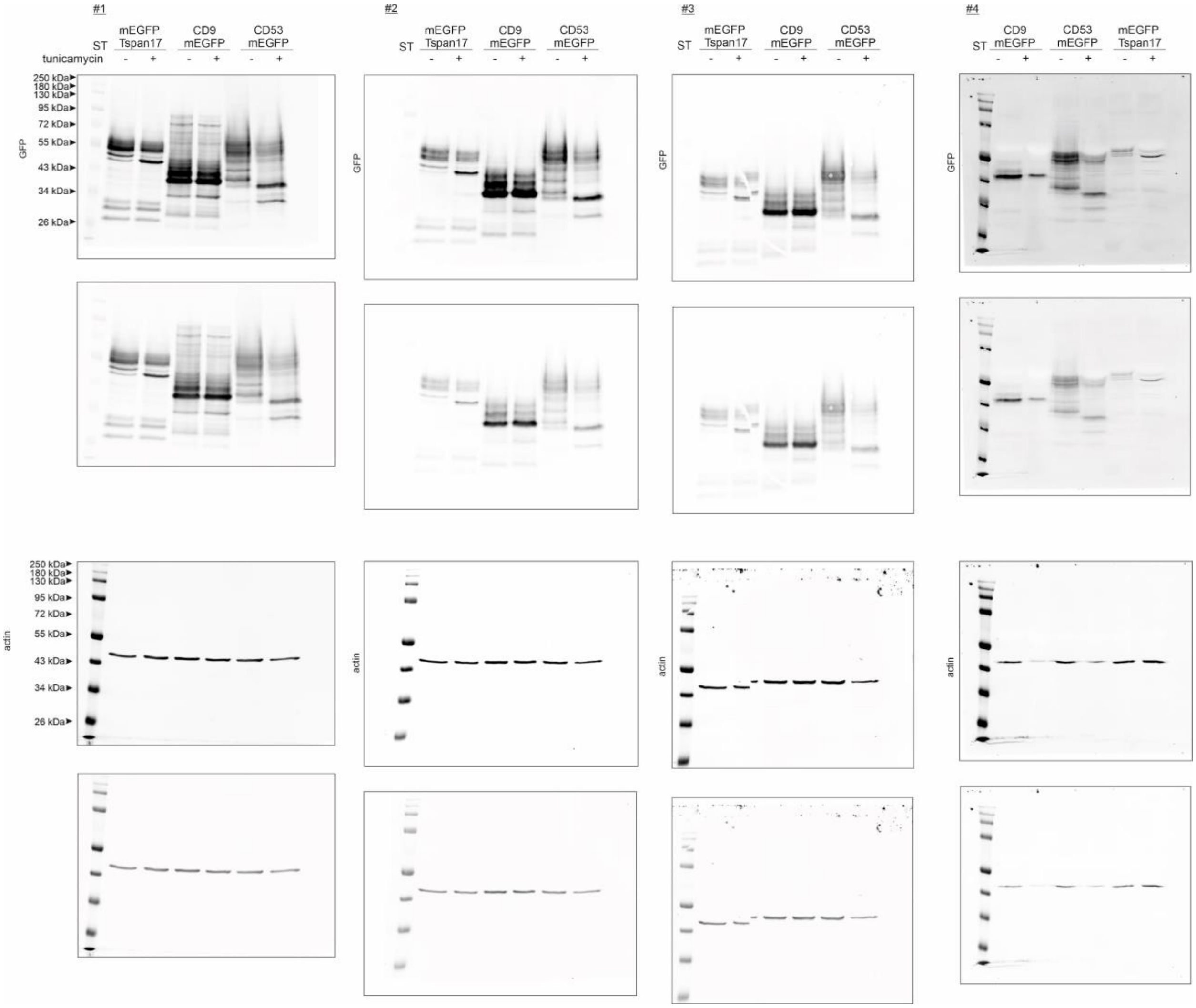


Figure S25: All blots used for the analysis in Figure S9
The four blots (#1-4) were stained for GFP (upper panel) and for actin (lower panel) and show the effect of tunicamycin treatment on the expression of mEGFP-Tspan17, CD9-mEGFP and CD53-mEGFP. They are shown with a protein standard (ST, 10-250 kDa range; NEB, #P7719S) and with two different scalings to ensure clear visibility of all bands. During blot imaging the focus lay on the area framed by the protein standard, therefore all blots used for this analysis (Fig. S9) are shown. The data illustration was performed using Fiji-ImageJ[9] (https://imagej.net/).

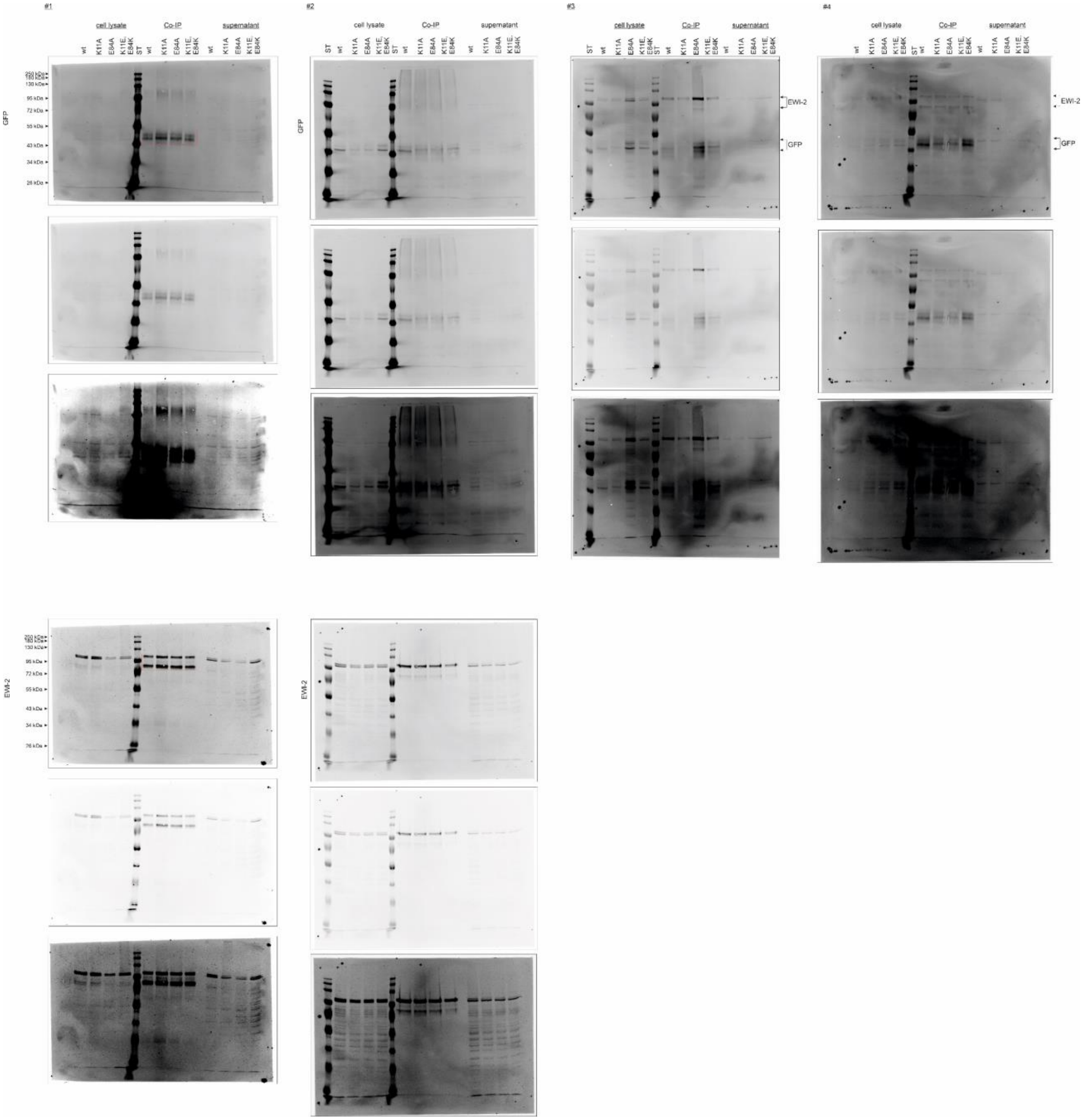


Figure S26: Full blot images used in Figure S13.

The four blots (#1-4) show the cell lysate, Co-Immunoprecipitation (Co-IP) and supernatant of the CD9-mEGFP salt-bridge mutations. The immunoprecipitation was stained for GFP (upper panel) and EWI-2 (lower panel). The EWI-2 molecule (upper band) is processed to EWI-2-Wint (lower band). The blots #3 and #4 show the GFP and EWI-2 staining in the same image, because they were stained using a secondary antibody attached to the same fluorescence dye (CW800). Therefore, the bands corresponding to GFP and EWI-2 staining are highlighted with arrows at the side of the blot. EWI-2-myc was transiently transfected and was stained via a EWI-2 antibody, which also detected the endogen levels and explains the double band. The blots are shown with a protein standard (ST, 10-250 kDa range; NEB, #P7719S) and with different scalings to ensure clear visibility of all bands. During blot imaging the focus lay on the area framed by the protein standard, therefore all blots used for this analysis (Fig. S13) are shown. The data illustration was performed using Fiji-ImageJ[9] (https://imagej.net/).

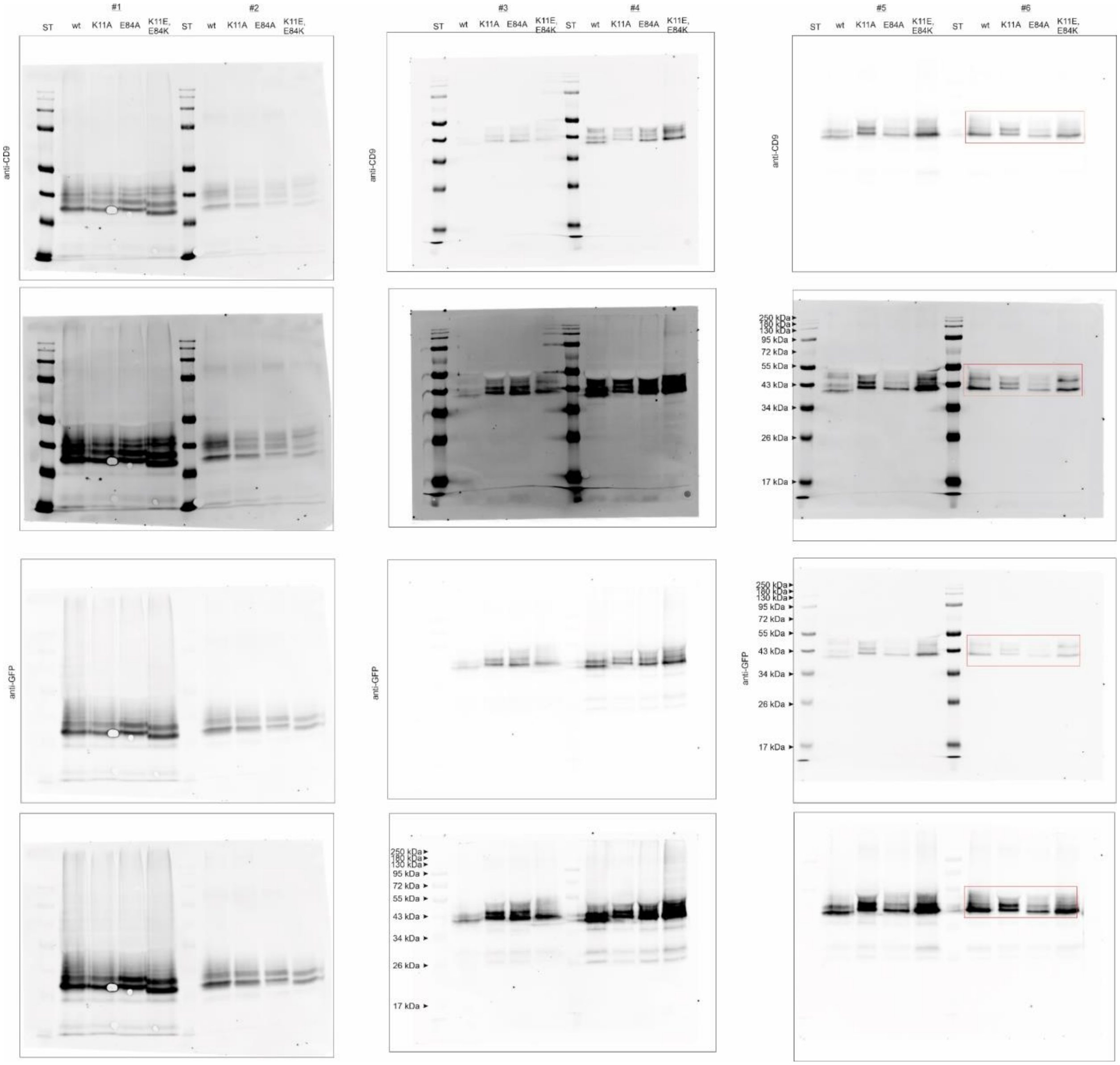


Figure S27: Full blot images used in Figure S16

The whole blots (#1-6 are the n=6) used for this analysis are shown in two different scalings and the cutout shown under (A) is highlighted by a red box in sample #6. The blots are shown with a protein standard (ST, 10-250 kDa range; NEB, #P7719S). The statistical analysis was done employing a repeated measures ANOVA comparing each mutation to the wild type (*P < 0.05, **P < 0.01, ***P < 0.001, ****P < 0.0001). The data analysis and illustration was performed using GraphPad Prism version 6.04 for Windows (www.graphpad.com) and Fiji-ImageJ[4] (https://imagej.net/).

## References


1. Dagona, A. G. BioEdit: a user-friendly biological sequence alignment editor and analysis program for Windows 95/98/NT. *Nucleic Acids Symp. Ser.* (1999).
2. Drozdetskiy, A., Cole, C., Procter, J. & Barton, G. J. JPred4: a protein secondary structure prediction server. *Nucleic Acids Res.* **43**, W389–W394 (2015).
3. Kyte, J. *Structure in Protein Chemistry*. (Garland Science, 2006).
4. Crooks, G. E., Hon, G., Chandonia, J.-M. & Brenner, S. E. WebLogo: A Sequence Logo Generator. 3.
5. Eisenberg, D. The discovery of the α-helix and β-sheet, the principal structural features of proteins. *Proc. Natl. Acad. Sci.* **100**, 11207–11210 (2003).
6. Krogh, A., Larsson, B., von Heijne, G. & Sonnhammer, E. L. Predicting transmembrane protein topology with a hidden Markov model: application to complete genomes. *J. Mol. Biol.* **305**, 567–580 (2001).
7. Sonnhammer, E. L., von Heijne, G. & Krogh, A. A hidden Markov model for predicting transmembrane helices in protein sequences. *Proc. Int. Conf. Intell. Syst. Mol. Biol.* **6**, 175–182 (1998).
8. Gautier, R., Douguet, D., Antonny, B. & Drin, G. HELIQUEST: a web server to screen sequences with specific alpha-helical properties. *Bioinforma. Oxf. Engl.* **24**, 2101–2102 (2008).
9. Schindelin, J. *et al.* Fiji: an open-source platform for biological-image analysis. *Nat. Methods* **9**, 676–682 (2012).
10. Gupta, R. & Brunak, S. Prediction of glycosylation across the human proteome and the correlation to protein function. *Pac. Symp. Biocomput. Pac. Symp. Biocomput.* 310–322 (2002).
11. Takayama, H., Chelikani, P., Reeves, P. J., Zhang, S. & Khorana, H. G. High-Level Expression, Single-Step Immunoaffinity Purification and Characterization of Human Tetraspanin Membrane Protein CD81. *PLoS ONE* **3**, e2314 (2008).
12. Zhu, Y.-Z. *et al.* Significance of palmitoylation of CD81 on its association with tetraspanin-enriched microdomains and mediating hepatitis C virus cell entry. *Virology* **429**, 112–123 (2012).
13. Seigneuret, M. Complete Predicted Three-Dimensional Structure of the Facilitator Transmembrane Protein and Hepatitis C Virus Receptor CD81: Conserved and Variable Structural Domains in the Tetraspanin Superfamily. *Biophys. J.* **90**, 212–227 (2006).
14. Cannon, K. S. & Cresswell, P. Quality control of transmembrane domain assembly in the tetraspanin CD82. *EMBO J.* **20**, 2443–2453 (2001).
